# Causal and Synthetic Associations of Variants in the *SERPINA* Gene Cluster with Alpha1-antitrypsin Serum Levels

**DOI:** 10.1371/journal.pgen.1003585

**Published:** 2013-08-22

**Authors:** Gian Andri Thun, Medea Imboden, Ilaria Ferrarotti, Ashish Kumar, Ma'en Obeidat, Michele Zorzetto, Margot Haun, Ivan Curjuric, Alexessander Couto Alves, Victoria E. Jackson, Eva Albrecht, Janina S. Ried, Alexander Teumer, Lorna M. Lopez, Jennifer E. Huffman, Stefan Enroth, Yohan Bossé, Ke Hao, Wim Timens, Ulf Gyllensten, Ozren Polasek, James F. Wilson, Igor Rudan, Caroline Hayward, Andrew J. Sandford, Ian J. Deary, Beate Koch, Eva Reischl, Holger Schulz, Jennie Hui, Alan L. James, Thierry Rochat, Erich W. Russi, Marjo-Riitta Jarvelin, David P. Strachan, Ian P. Hall, Martin D. Tobin, Morten Dahl, Sune Fallgaard Nielsen, Børge G. Nordestgaard, Florian Kronenberg, Maurizio Luisetti, Nicole M. Probst-Hensch

**Affiliations:** 1 Swiss Tropical and Public Health Institute, Basel, Switzerland; 2 University of Basel, Basel, Switzerland; 3 Center for Diagnosis of Inherited Alpha1-antitrypsin Deficiency, Institute for Respiratory Disease, IRCCS San Matteo Hospital Foundation, University of Pavia, Pavia, Italy; 4 Wellcome Trust Centre for Human Genetics, University of Oxford, Oxford, United Kingdom; 5 James Hogg Research Centre, Institute for Heart and Lung Health, University of British Columbia, Vancouver, Canada; 6 Division of Genetic Epidemiology, Department of Medical Genetics, Molecular and Clinical Pharmacology, Innsbruck Medical University, Innsbruck, Austria; 7 Department of Epidemiology and Biostatistics, Imperial College London, London, United Kingdom; 8 Departments of Health Sciences and Genetics, University of Leicester, Leicester, United Kingdom; 9 Institute of Genetic Epidemiology, Helmholtz Zentrum München - German Research Center for Environmental Health, Neuherberg, Germany; 10 Interfaculty Institute for Genetics and Functional Genomics, University Medicine Greifswald, Greifswald, Germany; 11 Centre for Cognitive Ageing and Cognitive Epidemiology, Department of Psychology, University of Edinburgh, Edinburgh, United Kingdom; 12 MRC Human Genetics, Institute of Genetics and Molecular Medicine, University of Edinburgh, Edinburgh, United Kingdom; 13 Department of Immunology, Genetics, and Pathology, Rudbeck Laboratory, SciLifeLab, Uppsala University, Uppsala, Sweden; 14 Institut Universitaire de Cardiologie et de Pneumologie de Québec, Department of Molecular Medicine, Laval University, Québec City, Canada; 15 Department of Genetics and Genomic Sciences, Icahn Institute of Genomics and Multiscale Biology, Icahn School of Medicine at Mount Sinai, New York, New York, United States of America; 16 Department of Pathology and Medical Biology, University Medical Center Groningen, GRIAC Research Institute, University of Groningen, Groningen, The Netherlands; 17 Department of Public Health, Faculty of Medicine, University of Split, Split, Croatia; 18 Centre for Population Health Sciences, Medical School, University of Edinburgh, Edinburgh, United Kingdom; 19 Department of Internal Medicine B, University Medicine Greifswald, Greifswald, Germany; 20 Research Unit of Molecular Epidemiology, Helmholtz Zentrum München - German Research Center for Environmental Health, Neuherberg, Germany; 21 Institute of Epidemiology I, Helmholtz Zentrum München - German Research Center for Environmental Health, Neuherberg, Germany; 22 School of Population Health, University of Western Australia, Perth, Australia; 23 Pathology and Laboratory Medicine, University of Western Australia, Perth, Australia; 24 Busselton Population Medical Research Foundation, Perth, Australia; 25 West Australian Sleep Disorders Research Institute, Perth, Australia; 26 School of Medicine and Pharmacology, University of Western Australia, Perth, Australia; 27 Division of Pulmonary Medicine, University Hospital of Geneva, Geneva, Switzerland; 28 Pulmonary Division, University Hospital of Zurich, Zurich, Switzerland; 29 Institute of Health Sciences, University of Oulu, Oulu, Finland; 30 Biocenter Oulu, University of Oulu, Oulu, Finland; 31 Unit of Primary Care, Oulu University Hospital, Oulu, Finland; 32 Department of Children and Young People and Families, National Institute for Health and Welfare, Oulu, Finland; 33 Division of Population Health Sciences and Education, St George's, University of London, London, United Kingdom; 34 Division of Therapeutics and Molecular Medicine, Queen's Medical Centre, Nottingham, United Kingdom; 35 Department of Clinical Biochemistry, Rigshospitalet, Copenhagen University Hospital, Copenhagen, Denmark; 36 Department of Clinical Biochemistry, Herlev Hospital, Copenhagen University Hospital, Herlev, Denmark; Georgia Institute of Technology, United States of America

## Abstract

Several infrequent genetic polymorphisms in the *SERPINA1* gene are known to substantially reduce concentration of alpha1-antitrypsin (AAT) in the blood. Since low AAT serum levels fail to protect pulmonary tissue from enzymatic degradation, these polymorphisms also increase the risk for early onset chronic obstructive pulmonary disease (COPD). The role of more common *SERPINA1* single nucleotide polymorphisms (SNPs) in respiratory health remains poorly understood.

We present here an agnostic investigation of genetic determinants of circulating AAT levels in a general population sample by performing a genome-wide association study (GWAS) in 1392 individuals of the SAPALDIA cohort.

Five common SNPs, defined by showing minor allele frequencies (MAFs) >5%, reached genome-wide significance, all located in the *SERPINA* gene cluster at 14q32.13. The top-ranking genotyped SNP rs4905179 was associated with an estimated effect of β = −0.068 g/L per minor allele (P = 1.20*10^−12^). But denser *SERPINA1* locus genotyping in 5569 participants with subsequent stepwise conditional analysis, as well as exon-sequencing in a subsample (N = 410), suggested that AAT serum level is causally determined at this locus by rare (MAF<1%) and low-frequent (MAF 1–5%) variants only, in particular by the well-documented protein inhibitor S and Z (PI S, PI Z) variants. Replication of the association of rs4905179 with AAT serum levels in the Copenhagen City Heart Study (N = 8273) was successful (P<0.0001), as was the replication of its synthetic nature (the effect disappeared after adjusting for PI S and Z, P = 0.57). Extending the analysis to lung function revealed a more complex situation. Only in individuals with severely compromised pulmonary health (N = 397), associations of common SNPs at this locus with lung function were driven by rarer PI S or Z variants. Overall, our meta-analysis of lung function in ever-smokers does not support a functional role of common SNPs in the *SERPINA* gene cluster in the general population.

## Author Summary

Low levels of alpha1-antitrypsin (AAT) in the blood are a well-established risk factor for accelerated loss in lung function and chronic obstructive pulmonary disease. While a few infrequent genetic polymorphisms are known to influence the serum levels of this enzyme, the role of common genetic variants has not been examined so far. The present genome-wide scan for associated variants in approximately 1400 Swiss inhabitants revealed a chromosomal locus containing the functionally established variants of AAT deficiency and variants previously associated with lung function and emphysema. We used dense genotyping of this genetic region in more than 5500 individuals and subsequent conditional analyses to unravel which of these associated variants contribute independently to the phenotype's variability. All associations of common variants could be attributed to the rarer functionally established variants, a result which was then replicated in an independent population-based Danish cohort. Hence, this locus represents a textbook example of how a large part of a trait's heritability can be hidden in infrequent genetic polymorphisms. The attempt to transfer these results to lung function furthermore suggests that effects of common variants in this genetic region in ever-smokers may also be explained by rarer variants, but only in individuals with hampered pulmonary health.

## Introduction

Alpha1-antitrypsin (AAT) is a serum marker for inflammation produced in the liver. Its main function is to inhibit neutrophil elastase and consequently protect pulmonary tissue. The *SERPINA1* gene encoding the AAT protein is known to be polymorphic in the general population. The best studied single nucleotide polymorphisms (SNPs) causing a reduction in AAT serum levels are the protease inhibitor S (PI S, rs17580) and the protease inhibitor Z (PI Z, rs28929474) variants [1]. The loss of function mechanism is especially well investigated for the PI Z variant. The resulting amino acid change in AAT leads to the protein's intracellular polymerization in hepatocytes and therefore to a reduced level of secreted serum AAT [2]. Homozygosity for PI Z (PI ZZ genotype) with a frequency of about 0.01% in Caucasian populations [3] causes blood AAT levels below 30% of normal. This genotype is clearly associated with elevated chronic obstructive pulmonary disease (COPD) risk accounting for 1–2% of all cases [4], [5]. There is also strong evidence that accelerated lung function decline and increased obstructive disease risk can be caused by compound heterozygosity of PI Z and PI S (PI SZ genotype). The case is less clear for PI MZ, PI MS or PI SS genotypes (PI M standing for the normal allele), which cause a less pronounced reduction in AAT concentration, as previous studies produced inconsistent evidence [6]–[9].

Further of note, large-scale genome-wide association studies (GWAS) on COPD or on cross-sectional or longitudinal lung function have not identified the *SERPINA1* gene to be a major genetic determinant [10]–[12]. But a recent GWAS on emphysema [13] and a comprehensive evaluation of candidate regions for lung function [14] reported rs4905179 and rs3748312, two common SNPs (minor allele frequencies (MAFs) >5%) located in the *SERPINA* gene cluster on 14q32.13, among their most strongly associated results. This locus encompasses *SERPINA1* and ten other genes (*SERPINA2* to *SERPINA6* and *SERPINA9* to *SERPINA13*) encoding extracellular ‘clade A’ serpins with very heterogeneous functions [15]. It is currently not known whether such association signals observed for this locus reflect a causal role of common variants or whether they are merely synthetic, reflecting effects of rarer causal variants [16]. Towards that aim, but also to detect further chromosomal loci of potential relevance to circulating levels of AAT, we first performed a GWAS on AAT serum level using a subset of the population-based Swiss Cohort Study of Air Pollution and Lung Disease in Adults (SAPALDIA) as discovery sample, and a second subset of SAPALDIA as well as an independent cohort, the Copenhagen City Heart Study (henceforth referred to as Copenhagen), as replication sample. We also conducted fine mapping analyses of the *SERPINA1* gene in the SAPALDIA cohort. Finally, we meta-analyzed the lung function effect of common and low-frequent *SERPINA1* SNPs previously observed to be associated with pulmonary health in ever-smokers, based on data provided by several population- and patient-based studies.

## Results

The discovery population and the design used to determine AAT-associated genetic variants are depicted in Figure S1 and further described in the Materials and Methods section. A comparison between the characteristics of the genome-wide analyzed sample (SAPALDIA discovery arm, N = 1392) and the remainder of the SAPALDIA cohort (SAPALDIA replication arm, N = 4245) did not reveal substantial differences in AAT serum levels or covariate distribution (Table S1), although asthmatics were overrepresented (39.4%) in the SAPALDIA discovery arm and absent in the replication arm, which is due to previous study design [17]. The participants of the independent replication cohort Copenhagen (N = 8273) were on average five years older and had twice as many current smokers (Table S1). This was in line with substantially lower lung function levels (more than 800 mL lower forced expiratory volume in 1 second, FEV1, compared to both SAPALDIA subsets) and slightly elevated AAT blood levels (1.339 g/L vs. 1.257 and 1.255 g/L, respectively). The characteristics of the study populations contributing to the genetic association analyses with lung function are given in Table S2.

### GWAS on AAT Serum Level

**Figure 1:**
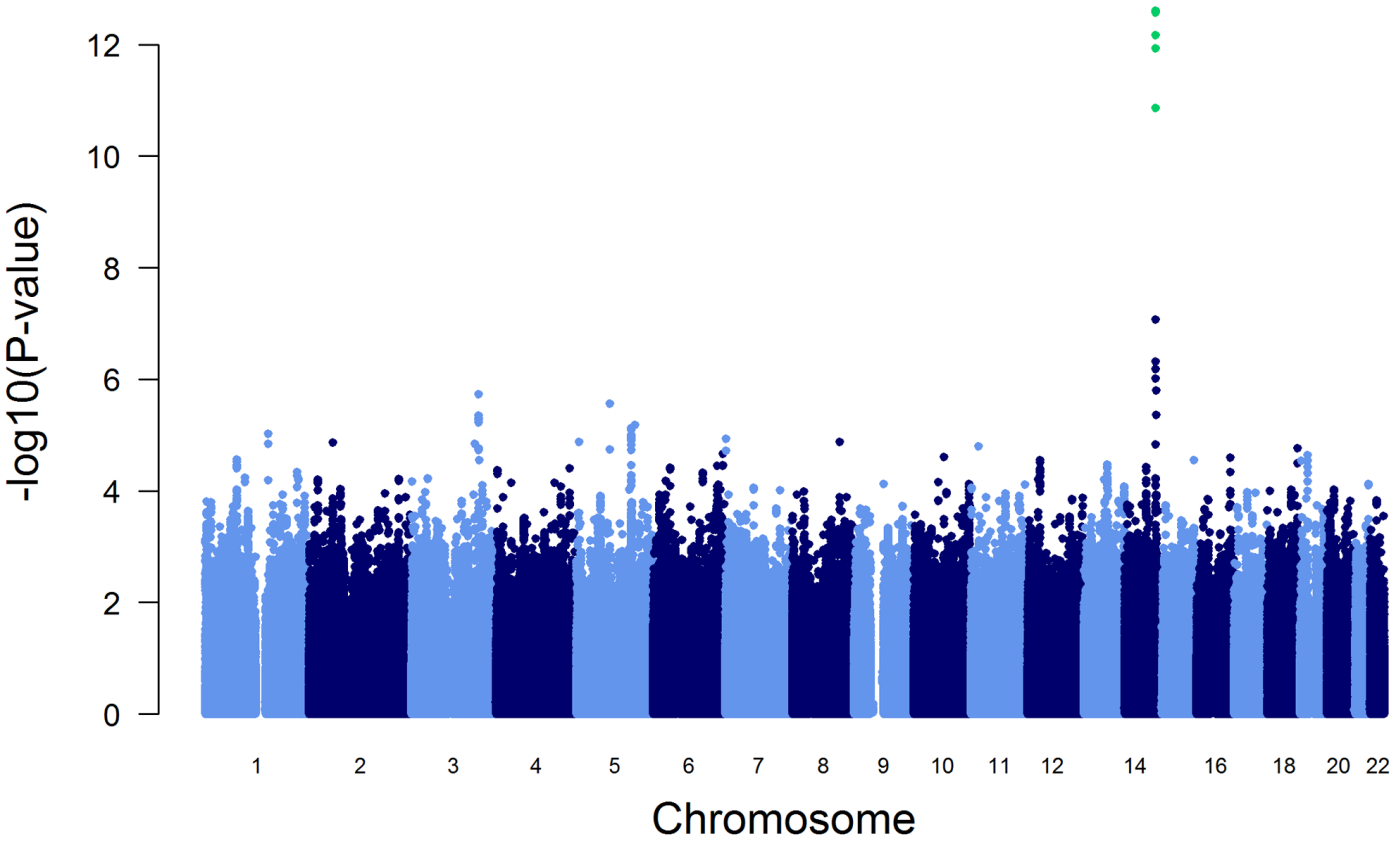
Manhattan plot of genome-wide -log(10) p-values for association with AAT serum level. SNPs reaching genome-wide significance are shown in green. They all belong to the *SERPINA* gene cluster.

**Figure 2:**
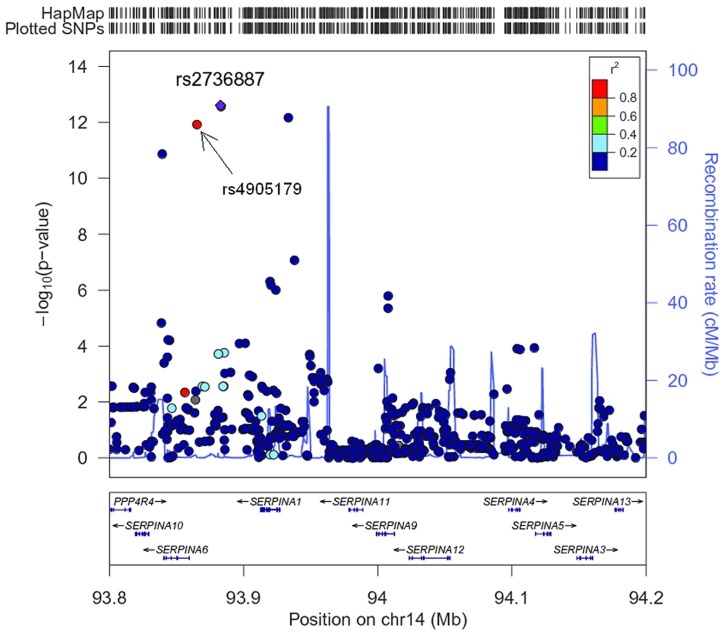
Regional plot for the *SERPINA* gene cluster (93.8–94.2 Mb on chromosome 14q32.13, reference panel: NCBI build 36.3). Presented are -log(10) p-values and LD (r^2^) with top-ranking SNP rs2736887 (purple diamond) for all SNPs in this region. The blue line shows recombination rate.

The association of more than 2.1 million genome-wide SNPs with AAT serum levels is shown in Figure 1. The ten most strongly associated SNPs were all located in the *SERPINA* gene cluster, half of them reached genome-wide significance (P<5*10^−8^, Table 1). The top 100 ranking SNPs are provided in Table S3. A regional association plot for the *SERPINA* gene cluster is shown in Figure 2. Both the top-ranking imputed SNP, rs2736887, and the top-ranking genotyped SNP, rs4905179, were located in close proximity to the *SERPINA6* gene and approximately 33 kb and 50 kb downstream of *SERPINA1* (effect estimates β = −0.071 and −0.068 g/L per minor allele; P = 2.48*10^−13^ and 1.20*10^−12^, respectively). Linkage disequilibrium (LD) between these two variants based on HapMap2 CEU (Utah residents with Northern and Western European ancestry) derived haplotype data [18] was strong (r^2^ = 0.88, D′ = 1), but Figure 2 suggests that the LD, expressed in r^2^ values, between the top-ranking SNP and the other SNPs in the region is generally modest. The genomic inflation factor lambda was low (λ = 1.02), suggesting minimal population stratification. The quantile-quantile plot (Q-Q plot) showed good adherence to null expectation and substantial positive deviation between observed and expected p-values for the top-ranking SNPs (Figure S2). In a sensitivity analysis adjusting for additional covariates, including high sensitivity C-reactive protein (hs-CRP), body mass index (BMI), passive smoking and alcohol intake, the genome-wide association results did not show an increase in the strength of the top-ranking loci, nor did they point to additional loci (data not shown). Even though this GWAS was enriched with asthma patients, GWAS stratification according to asthma status did not show heterogeneity for the top-ranking signals between participants with and without asthma (data not shown).

**Table 1:** The ten most strongly associated SNPs in the unconditional GWAS on AAT serum level in SAPALDIA (N = 1392).

SNP	Chromosome	Position	Gene	Location	Determination	MAF	Imp-r^2^	Allele Effect	P
rs2736887	14	93882733		intergenic	imputed	0.185	0.950	0.071	2.48E-13
rs926144	14	93883155		intergenic	imputed	0.186	0.950	0.071	2.72E-13
rs7151526	14	93933389	*SERPINA1*	5′UTR	imputed	0.065	0.769	0.116	6.78E-13
rs4905179	14	93865245	*SERPINA6*	5′UTR	genotyped	0.180	1.000	0.068	1.20E-12
rs11621961	14	93839229	*SERPINA6*	3′UTR	genotyped	0.355	0.945	0.052	1.37E-11
rs17751837	14	93937997	*SERPINA1*	5′UTR	genotyped	0.097	0.995	0.063	8.56E-08
rs1028580	14	93919635	*SERPINA1*	intron	imputed	0.154	0.979	0.051	4.87E-07
rs8010121	14	93920367	*SERPINA1*	intron	genotyped	0.155	0.999	0.049	6.64E-07
rs3748312	14	93924017	*SERPINA1*	intron	imputed	0.148	0.846	0.053	9.84E-07
rs17752593	14	94007781	*SERPINA9*	intron	genotyped	0.129	0.997	0.053	1.59E-06

Abbreviations: AAT, alpha1-antitrypsin; GWAS, genome-wide association study; MAF, minor allele frequency; SNP, single nucleotide polymorphism.

Imp-r^2^ is an indicator for imputation quality. SNPs with MAF<0.05 or imp-r^2^<0.5 were excluded.

Chromosomal position is based on reference panel, NCBI build 36.3. Allele effects are shown in absolute numbers.

### Association of 1000 Genomes Imputed Data for the *SERPINA* Gene Cluster with AAT Serum Level

In order to further refine association signals in this region, we imputed additional SNPs on chromosome 14 using haplotype data from the 1000 Genomes Project (1000G) [19]. The 1000G imputation yielded a three times higher number of imputed variants with reasonable quality scores (imputation-r^2^>0.5) compared to HapMap-derived imputed variants. A region defined by 1 Mb up- and downstream of the *SERPINA1* gene revealed 24 additional variants that were associated below a local significance level of P<3*10^−5^, adjusting for approximately 1800 SNPs covering a region of 2 Mb (Table S4). Among them, four low-frequent variants and one rare variant showed p-values reaching genome-wide significance level, and interestingly, none of them was in high LD (r^2^>0.8) with any other regional variant tested. The most strongly associated signal came from the PI Z variant, which is well known to be associated with reduced AAT serum levels (β = −0.620 g/L per minor allele, P = 4.61*10^−43^, MAF = 0.84%). The other well established causal polymorphism, the PI S variant, was less prominently ranked (β = −0.110 g/L per minor allele, P = 1.95*10^−6^, MAF = 5.70%) and exhibited an insufficient imputation quality (imputation-r^2^ = 0.45).

### GWAS on AAT Serum Level, Conditional on PI S and PI Z Variants

Accuracy of the imputed PI S and PI Z results was confirmed by direct genotyping of the samples [20]. The discovery arm revealed 33 PI Z carriers, 111 PI S carriers and two compound heterozygous carriers of PI S and PI Z (MAF = 1.26% for PI Z and 4.06% for PI S, respectively). No homozygous PI S or PI Z genotypes were detected.

**Figure 3:**
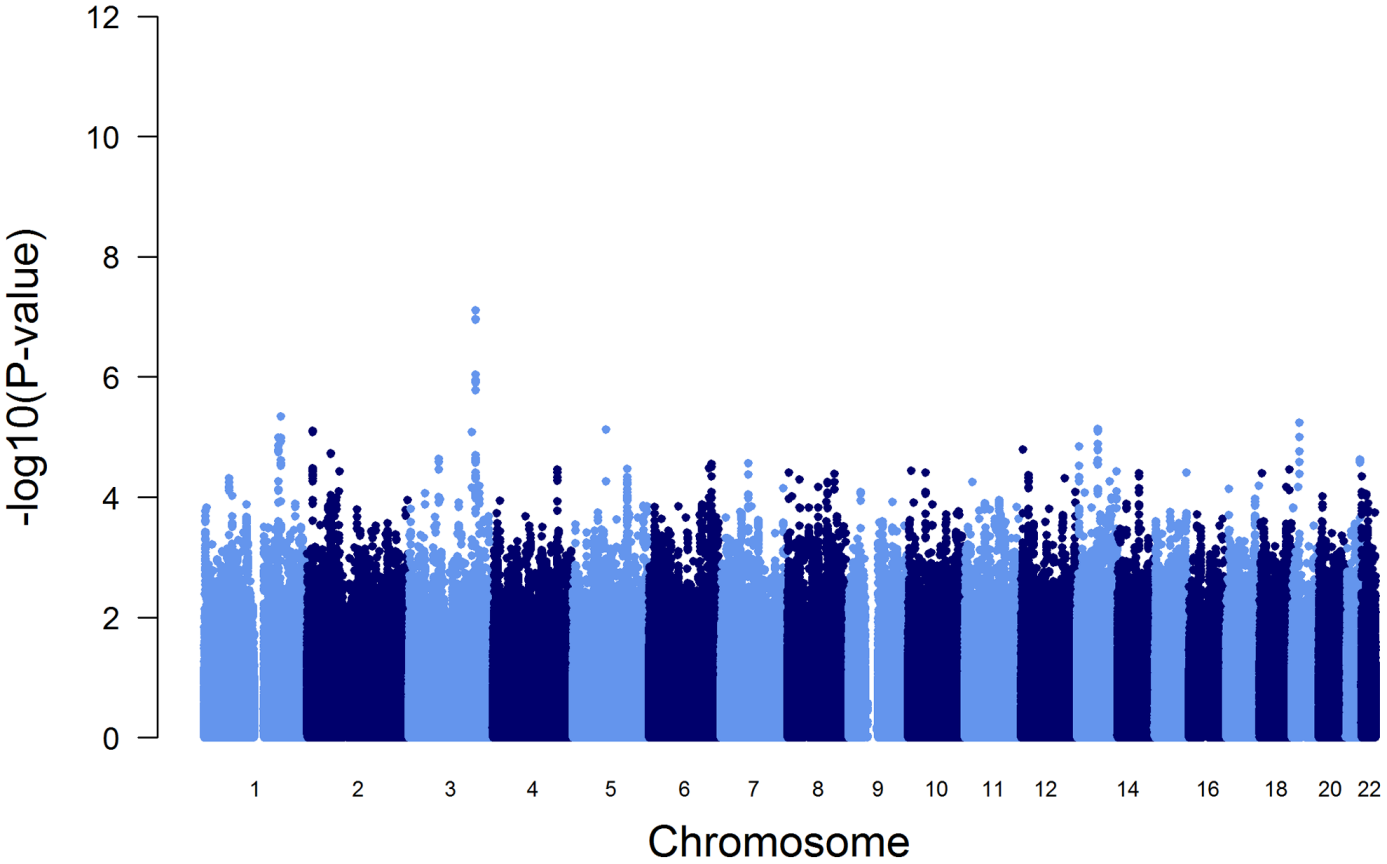
Manhattan plot of genome-wide -log(10) p-values for association with AAT serum level, conditional on PI S and PI Z alleles.

To test the influence of these variants on the initially reported GWAS results (Figure 1), we performed a conditional GWAS by additionally adjusting the regression models for the presence of PI S and PI Z alleles. We observed a drastic change in the association of the *SERPINA* gene cluster SNPs with AAT serum level (Figure 3 and Table 2). The strong signal on chromosome 14 observed in the original GWAS disappeared completely and the top-ranking imputed and genotyped SNPs (rs2736887 and rs4905179) were no longer significant (P = 0.44 and 0.31, respectively). In fact, no SNP was found near the *SERPINA* gene cluster among the 100 most strongly associated common variants (Table S5). In addition, the 1000G imputed data, comprising sequences 1 Mb up- and downstream of the *SERPINA1* gene, did not show evidence of other independent AAT-associated SNPs. An alternative approach that excluded all PI S and PI Z carriers from the GWAS sample (N = 146), instead of adjusting for them, confirmed the results. Both analyses revealed an intergenic region on chromosome 3 with borderline genome-wide significance (top-ranking SNP rs2566347, β = −0.043 g/L per minor allele, P = 7.88*10^−8^, in the adjusted GWAS). The top SNPs in this region were located in proximity to *MFSD1* and *RARRES1*, which are two genes with sparsely annotated function. The 1000G imputation of this region did not reveal further variants. In addition, we were unable to replicate this association signal in the SAPALDIA replication arm (N = 4245, β = −0.004 g/L per minor allele, P = 0.46).

**Table 2:** The ten most strongly associated SNPs in the GWAS on AAT serum level, conditional on PI S and PI Z alleles in SAPALDIA (N = 1392).

SNP	Chromosome	Position	Gene	Location	Determination	MAF	Imp-r^2^	Allele Effect	P
rs2566347	3	159974071		intergenic	imputed	0.192	0.998	0.043	7.88E-08
rs1560417	3	159972476		intergenic	imputed	0.200	0.998	0.042	1.11E-07
rs1560418	3	159972335		intergenic	genotyped	0.200	1.000	0.042	1.11E-07
rs1430414	3	159987697	*MFSD1*	5′UTR	imputed	0.137	0.984	0.045	9.26E-07
rs6761989	3	159983253		intergenic	imputed	0.137	0.993	0.044	1.14E-06
rs17643917	3	159968433		intergenic	imputed	0.137	1.000	0.044	1.23E-06
rs17643860	3	159967954		intergenic	imputed	0.137	1.000	0.044	1.24E-06
rs17700475	3	159967627		intergenic	genotyped	0.137	1.000	0.044	1.25E-06
rs3863076	3	159969394		intergenic	genotyped	0.145	1.000	0.042	1.69E-06
rs2206593	1	184909052	*PTGS2*	3′UTR	genotyped	0.065	0.956	0.060	4.60E-06

Abbreviations: AAT, alpha1-antitrypsin; GWAS, genome-wide association study; MAF, minor allele frequency; SNP, single nucleotide polymorphism.

Imp-r^2^ is an indicator for imputation quality. SNPs with MAF<0.05 or imp-r^2^<0.5 were excluded.

Chromosomal position is based on reference panel, NCBI build 36.3. Allele effects are shown in absolute numbers.

### Replication in the Copenhagen City Heart Study

The effect of rs4905179, the top genotyped SNP in our GWAS, on AAT serum levels was tested for replication in Copenhagen (Table 3). The minor allele was associated with β = −0.097 g/L (P<0.0001, N = 8332). As observed in the GWAS, adjustment for PI S and Z polymorphisms resulted in a complete loss of this signal (β = 0.003 g/L, P = 0.57, N = 8273).

**Table 3:** Minor allele effects of PI S, PI Z and rs4905179 on AAT serum levels in the Copenhagen City Heart Study.

SNP	N	MAF (genotyped)	Allele Effect (g/L)	95% Confidence Intervals	P
rs17580 (PI S)	8338	0.029	−0.188	−0.211 to −0.165	<0.0001
rs28929474 (PI Z)	8338	0.027	−0.492	−0.514 to −0.470	<0.0001
rs4905179	8332	0.186	−0.097	−0.107 to −0.087	<0.0001
rs4905179, adjusted for PI S and Z	8273	0.186	0.003	−0.007 to 0.013	0.57

Abbreviations: MAF, minor allele frequency; SNP, single nucleotide polymorphism.

### Impact of Common and Low-Frequent *SERPINA1* Genetic Variants on AAT Serum Level

**Figure 4:**
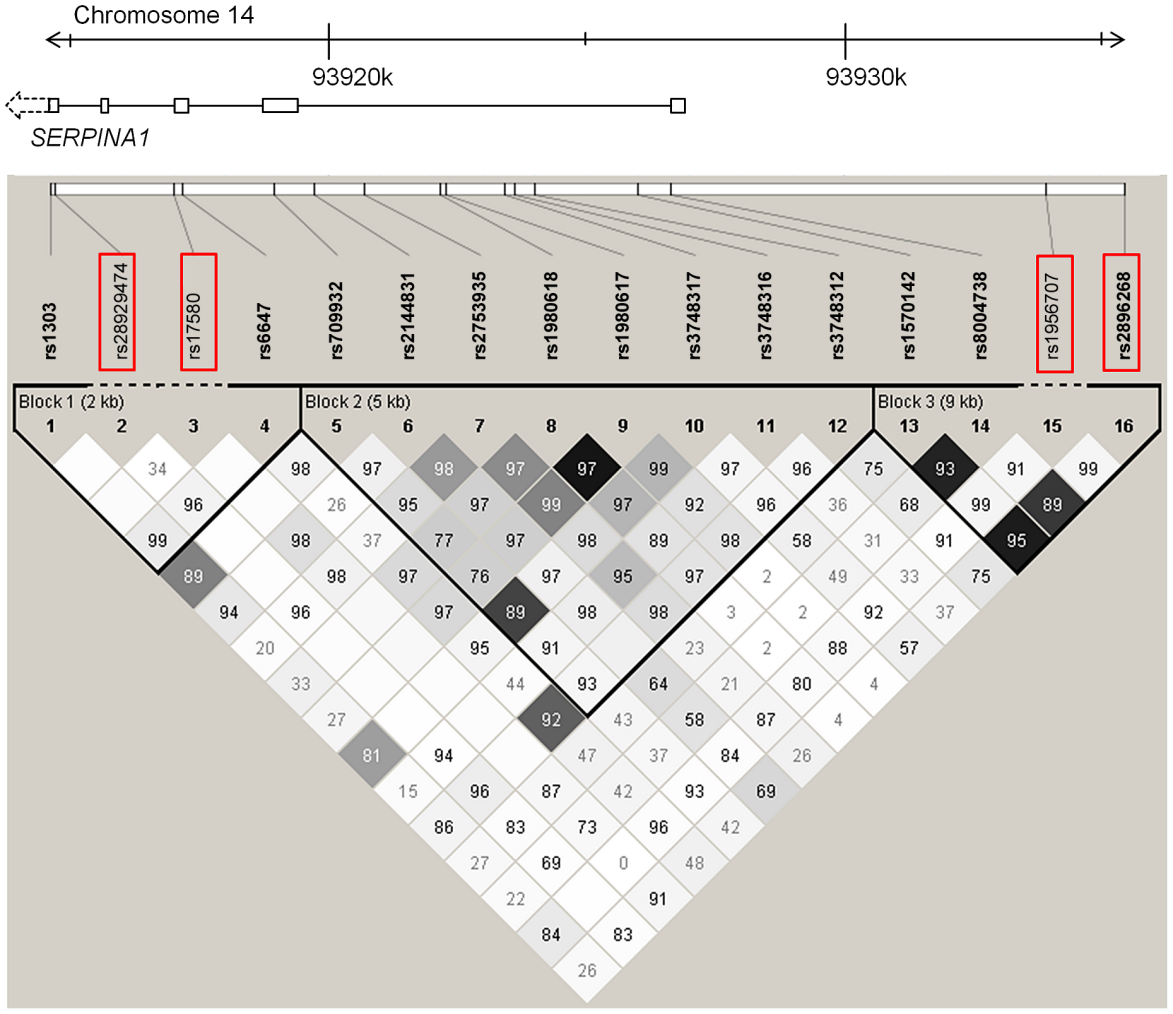
LD plot among common and low-frequent SNPs in the *SERPINA1* gene within the SAPALDIA study. Shading represent r^2^ values, whereas numbers represent D′ values (no number equals D′ = 1). Red framed SNPs are independently associated with AAT serum levels after forward selection stepwise regression modeling. Rs17580 is the PI S variant and rs28929474 is the PI Z variant.

In a first fine mapping step, 16 *SERPINA1* SNPs (see Materials and Methods section for a description of the SNP selection) were successfully genotyped in 5569 SAPALDIA subjects (discovery and replication arm combined). The genotype results in the discovery arm allowed us to compare allele frequencies with imputed results derived from the 1000G data. Table S6 shows that the agreement was very high. Stepwise conditional regression analyses were then applied to evaluate the independent effects of each of these SNPs on AAT serum levels (Table 4). The PI Z variant was most strongly associated with circulating levels of AAT. The PI S variant remained strongly associated after conditioning on PI Z. Two variants located in the 5′ non-coding gene region (rs2896268 and rs1956707) were marginally associated with the phenotype in two further steps after conditioning on PI S and PI Z. The total variance of AAT explained by statistical models increased from 8.8% (model with only non-genetic factors) to 32.6% (adding PI S and PI Z alleles), and to 32.8% adding rs2896268 and rs1956707. Based on genotype data from the SAPALDIA cohort, the *SERPINA1* gene contains three haplotype blocks using D′-based block definition (Figure 4). The AAT deficiency variants PI S and PI Z are located in block 1, while rs2896268 and rs1956707 are located in block 3, roughly 8 kb upstream of exon 1.

**Table 4:** Common and low-frequent *SERPINA1* SNPs and their association with AAT serum level, univariate and conditional on significantly associated SNPs (N = 5569^a^), in SAPALDIA.

SNP	Location	Position	Selection^b^	MAF	Univariate^c^		Conditional^d^	
					Allele Effect	P	Allele Effect	P
rs2896268	5′UTR	93935461	C	0.495	0.006	0.10	**0.013**	**4.1E-05**
rs1956707	5′UTR	93933946	C	0.038	0.016	0.10	**0.029**	**5.0E-04**
rs8004738	exon 1	93926667	D	0.490	0.005	0.15	0.001	0.81
rs1570142	intron 1	93926015	A,B,C	0.488	0.005	0.19	0.001	0.88
rs3748312	intron 1	93924017	A,B	0.153	0.035	2.4E-12	0.002	0.70
rs3748316	intron 1	93923617	A,C	0.181	0.011	0.02	0.002	0.63
rs3748317	intron 1	93923432	A	0.158	0.020	5.5E-05	0.004	0.36
rs1980617	intron 1	93922287	A	0.389	0.030	5.0E-16	0.003	0.31
rs1980618	intron 1	93922176	A,C	0.383	0.030	1.3E-16	0.005	0.15
rs2753935	intron 1	93920690	A	0.435	0.012	9.7E-04	0.004	0.24
rs2144831	intron 1	93919723	A,C	0.240	0.021	9.8E-07	0.002	0.71
rs709932	exon 2	93918954	A,C	0.169	0.021	1.2E-05	0.003	0.50
rs6647	exon 3	93917168	A,C	0.200	0.026	7.2E-09	0.003	0.51
rs17580 (PI S)	exon 3	93917015	D	0.041	0.210	1.9E-127	**0.218**	**1.9E-163**
rs28929474 (PI Z)	exon 5	93914700	D	0.013	0.483	1.3E-240	**0.482**	**5.2E-269**
rs1303	exon 5	93914596	A,C	0.247	0.015	4.7E-04	0.001	0.89

Abbreviations: AAT, alpha1-antitrypsin; MAF, minor allele frequency; SNP, single nucleotide polymorphism.

Chromosomal position is based on reference panel, NCBI build 36.3.

a Includes subjects for whom all the 16 SNPs have been successfully genotyped.

b SNP selection was based on extreme trait sequence data (A), tagging SNPs according to HapMap (B), TAMAL software (C) and publication about functionality (D); see Materials and Methods for a more detailed description.

c Univariate analyses were adjusted for non-genetic factors only (sex, age, recruiting area and current smoking status). Allele effects are shown in absolute numbers and P<0.005 was considered statistically significant.

d In a forward selection approach of stepwise regression, the four SNPs in bold contributed statistically significantly to the variability in AAT serum levels and were included in the final statistical model. Allele effects and p-values refer to this final model.

### Impact of Rare *SERPINA1* Genetic Variants on AAT Serum Level

In a second fine mapping step, exon sequencing was performed in 410 subjects with low AAT levels that were independent of the presence of PI S or PI Z alleles [21]. 16 additional *SERPINA1* variants (two deletions and 14 SNPs) were detected, of which all but one had already been described [21]–[29] (Table S7). Three of the SNPs were synonymous, and five had no accession numbers in public databases (as of April 1^st^, 2013). Most of the non-synonymous SNPs have already been described as potentially lowering AAT serum level, and computational tools only classified one of them as no damaging to the protein's tertiary structure. In order to estimate the phenotypic influence of these rare variants, we compared mean AAT blood levels, adjusted for sex, age, study center, current smoking, as well as for the presence of PI S and Z alleles, between samples without rare variants (N = 346) and those with a single rare variant (N = 63) or more than one (N = 1). The subjects with rare variants had a lower adjusted mean AAT level (0.904 g/L, 95% CI 0.884 to 0.924 g/L) compared to those without rare variants (0.992 g/L, 95% CI 0.984 to 1.000 g/L, P<0.001). Although this difference is small, the range covers the recently proposed upper limit of intermediate AAT deficiency (0.92 g/L), a value with some clinical relevance [20]. AAT levels of carriers of synonymous mutations or non-synonymous mutations without predicted damaging consequences to protein structure (N = 20) were not different from those carrying no rare variants (0.985 vs. 0.990 g/L, P = 0.77). Assuming that unsequenced samples were negative for mutations with predicted deleterious functional effects, the total variance of explained AAT further increased from 32.8% to 35.4% (based on a statistical model adding all rare mutations with predicted damaging consequences to the protein structure).

### Common and Low-Frequent *SERPINA1* SNPs Previously Associated with Lung Function

**Figure 5:**
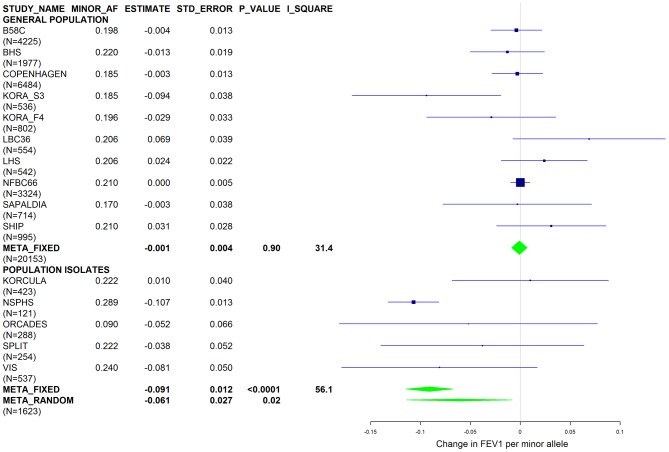
Forest plot of meta-analyzed results for the effect per minor allele of rs4905179 on FEV1 in ever-smokers, adjusted for sex, age, height and population stratification factors. Studies based on population isolates with a high degree of cryptic relatedness are presented separately. Effect estimates of meta-analyses are shown with green diamonds. I^2^ is a measure of the heterogeneity between studies. Random effect meta-analyses are included if I^2^>0.5. Study weights (blue squares) correspond to fixed effect meta-analyses.

**Figure 6:**
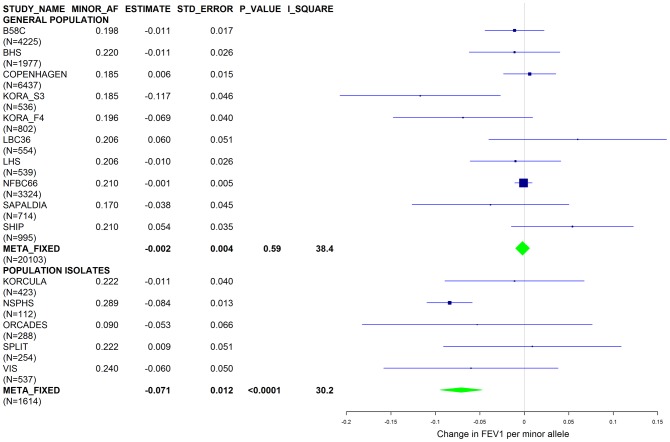
Forest plot of meta-analyzed results for the effect per minor allele of rs4905179 on FEV1 in ever-smokers, adjusted for sex, age, height, population stratification factors and the presence of PI S and Z alleles. Studies based on population isolates with a high degree of cryptic relatedness are presented separately. Effect estimates of meta-analyses are shown with green diamonds. I^2^ is a measure of the heterogeneity between studies. Random effect meta-analyses are included if I^2^>0.5. Study weights (blue squares) correspond to fixed effect meta-analyses.

**Figure 7:**
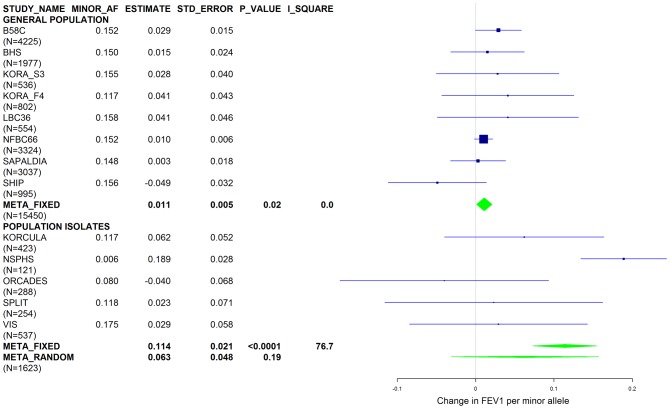
Forest plot of meta-analyzed results for the effect per minor allele of rs3748312 on FEV1 in ever-smokers, adjusted for sex, age, height and population stratification factors. Studies based on population isolates with a high degree of cryptic relatedness are presented separately. Effect estimates of meta-analyses are shown with green diamonds. I^2^ is a measure of the heterogeneity between studies. Random effect meta-analyses are included if I^2^>0.5. Study weights (blue squares) correspond to fixed effect meta-analyses.

**Figure 8:**
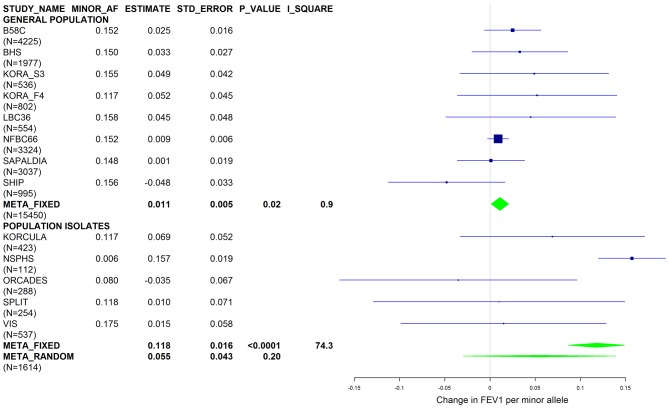
Forest plot of meta-analyzed results for the effect per minor allele of rs3748312 on FEV1 in ever-smokers, adjusted for sex, age, height, population stratification factors and the presence of PI S and Z alleles. Studies based on population isolates with a high degree of cryptic relatedness are presented separately. Effect estimates of meta-analyses are shown with green diamonds. I^2^ is a measure of the heterogeneity between studies. Random effect meta-analyses are included if I^2^>0.5. Study weights (blue squares) correspond to fixed effect meta-analyses.

Results from a previous GWAS on emphysema [13] and a large-scale evaluation of candidate loci on lung function [14] pointed to a role of common variants in the *SERPINA* gene cluster. The SNPs rs4905179 (associated with emphysema in smokers [13]) and rs3748312 (associated with cross-sectional lung function among ever smokers [14]) were strongly associated with AAT in our study (Tables 1 and 4), but both signals disappeared upon adjustment for the low-frequent variants PI S and Z. In order to clarify whether the association of the two common SNPs with pulmonary health could also be explained by effects of the rarer SNPs, we conducted a meta-analysis for cross-sectional lung function in ever-smokers across 17 studies with a total sample size of N = 24,446 (Table S2). We included nine studies which had contributed to the original finding on lung function [14] and had available genotypes or 1000G imputed genotype data on PI S and Z. The meta-analysis in cohorts of general population study design showed that rs4905179 was not associated with lung function in ever-smokers (P = 0.90 in the fixed-effect meta-analysis, N = 20,153, Figure 5). Yet smaller studies recruited within population isolates showed a trend for the rare allele to be associated with low lung function (random-effect P = 0.02, N = 1623, Figure 5), and in contrast to the association with AAT serum levels, adjusting for PI S and Z alleles did not modify the association of rs4905179 with lung function (Figure 6). For the second common SNP, rs3748312, we could nominally replicate the statistically significant allele effect on FEV1 in the general population of ever-smokers (P = 0.02, N = 15,450), and the stronger effect that was published [14] seems to be driven by population isolates (Figure 7). Again, as for rs4905179, the associations were not dependent on S and Z alleles (Figure 8). Meta-analyses of the associations of PI S and Z alleles with lung function revealed no consistent associations between these functional AAT level determining variants and reduced FEV1 (Figures S3 and S4). Remarkably, the significant associations of rs4905179 and rs3748312 with lung function assessed in two additional studies with patients featuring compromised pulmonary health and undergoing lung resection, showed evidence for synthetic associations of the common SNPs with lung function that are consistent with our results for circulating AAT (Table 5). The minor alleles were associated with lower lung function and the association completely disappeared when conditioned on the presence of PI S and Z alleles.

**Table 5:** Minor allele effects on FEV1 of low-frequent and common SNPs in the *SERPINA* gene cluster in ever-smokers undergoing lung resection.

SNP	MAF	Imp-r^2^	Allele Effect (L)	P	MAF	Imp-r^2^	Allele Effect (L)	P
	Groningen (N = 133)				University of British Columbia, UBC (N = 264)			
rs17580 (PI S)	0.055	0.72	0.42	0.10	0.079	0.76	−0.15	0.29
rs28929474 (PI Z)	0.123	0.98	−0.68	<0.001	0.032	0.98	−0.81	<0.0001
								
rs4905179	0.299	1.00	−0.22	0.04	0.249	na^a^	−0.23	0.002
rs4905179, adjusted for PI S and Z			−0.07	0.65			−0.08	0.36
rs3748312	0.233	0.95	−0.34	0.008	0.148	0.94	−0.16	0.07
rs3748312, adjusted for PI S and Z			0.16	0.37			0.09	0.35

Abbreviations: FEV1, forced expiratory volume in one second; MAF, minor allele frequency; SNP, single nucleotide polymorphism.

Imp-r^2^ is an indicator for imputation quality. The analyses were adjusted for age, sex and height.

a Rs4905179 was genotyped in UBC.

## Discussion

We present here the first GWAS on circulating AAT blood levels. Our results confirm that genetic variation in the *SERPINA1* gene is a strong determinant of serum AAT levels. Fine mapping of *SERPINA1* and subsequent stepwise regression analyses further revealed that the associations with common variants in the *SERPINA* locus could be attributed to rarer variants previously identified to be causally linked with AAT deficiency.

There is an ongoing debate about whether rare variants are responsible for the missing heritability observed in GWAS on many complex outcomes [30]. We show here an example in which the polymorphisms PI S and PI Z seem to account for basically all observable effects of common variants in the *SERPINA* gene cluster on AAT serum level. The top-ranking genotyped SNP in our GWAS, rs4905179, was in low r^2^-based LD with PI S (r^2^ = 0.18) and PI Z (r^2^ = 0.06), reflecting in part the unequal allele frequencies of these SNPs. However, PI S and PI Z showed very high LD in terms of D′ with the GWAS top signals (e.g. D′ = 0.95 and 0.96, respectively, with rs4905179) and generally with many common variants in this locus (Figure 4), suggesting little genetic recombination. This proof-of-principle approach, revealing that signals of common variants in fact merely reflect rarer variants, has recently also been shown for some of the loci regulating low-density lipoprotein (LDL) cholesterol [31], [32]. Yet for other loci linked to LDL cholesterol, as well as for loci influencing other traits, both common and low-frequent variants contributed independently of the original GWAS signal to the phenotypic trait [31], [33], [34].

Using regional 1000G imputation within the top-ranking loci can allow the identification of additional association signals of stronger size to support the initial GWAS top result, as observed here for the *SERPINA* cluster, but not for the locus near *MFSD1*, an association which was not confirmed in the SAPALDIA replication arm. The resequencing strategy of the GWAS-identified locus in a sample with low AAT concentrations yielded in the identification of rare variants being strongly associated with reduced AAT blood levels. Such an accumulation of rare variants in the extreme range of the respective phenotype has also been reported by others [35], [36]. As for the relative contribution of genetic variants on the phenotype, we confirmed that effect sizes of PI S and Z on AAT serum levels were comparably strong, explaining alone a high proportion of the total variability (24.2%). We estimated that rare variants explained at least another 2% in our population-based sample, but since we did not sequence the entire SAPALDIA sample for rare variants, we cannot reliably quantify this contribution. In terms of blood markers, similar examples exist in which one genetic variant could explain well above 5% of the phenotype's variability (e.g. lipoprotein(a) [37], bilirubin [38] or adiponectin [39]), but for many other markers like serum lipid levels, only variants with small effects have been detected so far [40].

Association patterns between *SERPINA1* variants and circulating AAT did not translate to according associations with lung function level in a straightforward manner. Lung function is a complex phenotype associated with numerous genetic variants [11], [12]. Studies on the associations of *SERPINA1* polymorphisms with lung function and COPD have produced mixed results. It is well accepted that severe AAT deficiency caused by PI null mutations or by the presence of two PI Z alleles puts a subgroup of carriers at higher risk of emphysema and COPD, especially when smoking [5]. Studies on COPD found suggestive evidence for an association with heterozygous status for the PI Z allele [6], [8], but we observed no associations in our meta-analysis between PI Z and lung function level in ever-smokers. In the SAPALDIA general population sample, we had previously reported that an effect of the PI Z allele on lung function decline is restricted to persistent smokers and primarily observed for forced expiratory flow 25–75% [9]. Other variation in or close to the *SERPINA1* gene has been proposed to play a role for pulmonary health. First, a haplotype pattern of five common SNPs was reported to be more frequent in COPD cases than in controls in a study with limited statistical power [41]. The only SNP which was also separately associated with COPD in that analysis was not associated with reduced serum levels in our study (rs8004738, Table 4). Second, the minor allele of rs4905179, which was the top signal in the current AAT GWAS, was positively associated with emphysema assessed by chest tomography in three independent cohorts consisting of smoking COPD patients without severe AAT deficiency (PI ZZ) [13]. Finally, the minor allele of the intronic SNP rs3748312 was positively associated with lung function in ever-smokers from different population-based studies of the SpiroMeta Consortium [14]. The association of these two SNPs with lung function in ever-smokers was heterogeneous across studies in our meta-analysis. Dependency on PI S and Z was limited to studies in patients with lung resection (Groningen, UBC), consistent with the notion that *SERPINA1* may only confer risk in selected population subgroups.

There are several possible explanations for the poor translation of genetic association patterns with serum AAT to lung function and for the heterogeneity of associations between *SERPINA1* variants and lung function. First, lung function is influenced by mechanisms in addition to protease-antiprotease disequilibrium. Second, the contribution of the *SERPINA1* gene variants as a determinant of lung function likely depends on both the evolutionary pressure in isolated populations and the prevalence of effect modifiers in the respective study populations. These include smoking and smoking intensity, and likely other markers of inflammation. AAT itself plays a dual role in its relationship with lung function. While chronic AAT deficiency is etiologically associated with adverse pulmonary health, individuals with lung function impairment in fact exhibit higher AAT levels for a given genetic background due to AAT's role as an acute-phase inflammation marker [42], [43]. Third, tissue-specific regulation of the *SERPINA1* locus may play an important role. Serum AAT levels are driven by *SERPINA1* expression, protein formation and secretion in hepatocytes, so that regulatory SNPs associated with serum AAT likely reflect processes in the liver. One way to infer causality of potentially regulatory SNPs is by testing if they are simultaneously associated with health outcome and gene expression in the relevant tissue [44]. We therefore conducted a look-up in an expression quantitative loci (eQTL) database of lung tissue [45], but could not find any common variant which was significantly associated in *cis* with the transcripts deriving from the *SERPINA1* locus. In a recent study on networks of blood metabolites, the SNPs rs11628917 and rs1884549 were the most strongly associated blood and liver eQTLs with respect to *SERPINA1* expression [46]. They both lie in the 3′ untranslated region of *SERPINA1*, but were not associated with blood AAT in our GWAS (P = 0.80 and P = 0.21, respectively). Moreover, we could not detect epistasis between those variants and the deleterious coding variants PI S and Z in terms of AAT serum levels. The absence of such an interaction does not point to regulatory function of the common SNPs [47] and argues in favor of tissue-specific heterogeneity. Forth, the role of *SERPINA1* in selected subgroups of persons exhibiting accelerated lung function decline or COPD needs to be considered from a perspective beyond genetic variation, as a recent study investigating epigenetic mechanisms of disease revealed methylation status of the *SERPINA1* gene to be most strongly associated with cross-sectional lung function and COPD [48].

The strength of this study is that it combines the report of a GWAS on AAT serum levels with meta-analyses of the associations of some of the GWAS top variants with lung function. The effects of the underlying functional variants are thoroughly investigated resulting in the hitherto largest meta-analysis of PI S and Z on FEV1 in ever-smokers. Ascertainment and study design of the many participating studies were sufficiently diverse to informatively address heterogeneity in association of common and rarer variants in the *SERPINA* gene cluster with lung function. The strength of the discovery sample is the population-based study design and the detailed characterization of the participants. Sex, age and smoking are important modifiers of AAT blood levels in the general population [43] and were included in all regression models. More refined smoking variables covering smoking intensity were not included as this information is less complete than smoking status in SAPALDIA and would lower the sample size. By excluding samples with elevated hs-CRP values we avoided the masking of AAT deficiencies due to a chronic or acute inflammation. On the methodological side, conditional analysis is a well-established tool for identifying independent signals within a certain locus [38], [49], [50]. Furthermore, 1000G imputation was able to point to the causal variant demonstrating its reliability to correctly assign alleles close to the 1% MAF threshold.

The limitations of this investigation include firstly the small sample size of the GWAS discovery arm, resulting in a high susceptibility to false negative findings. We calculated 63% power to detect SNPs with an allele effect of 0.1 g/L AAT serum level ( = 2.4% of the phenotypic variance) to a genome-wide significance level of 5*10^−8^. However, if we define the clinically important threshold of AAT as the upper limit of intermediate AAT deficiency, which has been recently suggested as 0.92 g/L [20], we have more than 99.9% power to detect such a large-impact variant. Nevertheless, genes that contribute to AAT serum levels with smaller effects than *SERPINA1* were likely to be missed. This could be a reason why neither SNPs in interleukin 6 (IL-6) nor in hepatocyte nuclear factor 1α (HNF-1α)/HNF-4, both important regulators of AAT expression [51], were associated with circulating AAT concentrations. Furthermore, by sequencing only the coding region of *SERPINA1*, rare variants in introns and outside the gene could not be determined. Another potential limitation of our GWAS on AAT serum level is the overrepresentation of asthmatics in the discovery sample. Asthma patients usually show higher levels of inflammatory markers in their lungs. However, we did not find heterogeneity in the effects of the most strongly associated SNPs when comparing asthmatics with non-asthmatics. Moreover, AAT mean values between the discovery and the replication arm were not significantly different, as participants with elevated hs-CRP had been excluded.

In conclusion, our study confirms the *SERPINA1* locus as the major genetic determinant of AAT blood levels. Methodologically, it represents a powerful example how low-frequent variants, separated by several kilobases from the top-ranking GWAS signals, can create purely synthetic associations which do not add to the variance of the respective outcome. In terms of lung function, our data do not support a functional role of any common SNP in the *SERPINA* cluster in the general population.

## Materials and Methods

### Ethics Statement

SAPALDIA was approved by the Swiss Academy of Medical Sciences, the national ethics committee for clinical research (UREK, Project Approval Number 123/00) and the Cantonal Ethics Committees for each of the eight examination areas (Ethics commissions of the cantons Aargau, Basel, Geneva, Grisons, Ticino, Valais, Vaud and Zurich). Participants were required to give written consent before any part of the health examination was conducted either globally (for all health examinations) or separately for each investigation. For ethics statements of the additional studies contributing to this work, see Table S8.

### Study Population

#### SAPALDIA

In 1991, a random sample of 9651 adults, aged 18–60 years, from eight areas in Switzerland responded to a questionnaire about respiratory health, occupational and lifestyle exposures. 99.0% of them also underwent spirometry testing [52]. Eleven years later, 8047 persons were reassessed and 6058 subjects provided blood samples and consented to DNA analysis [53]. In the present study, we used a subgroup of the second survey (N = 1640) that underwent genotyping in the context of the GWAS on asthma by the GABRIEL consortium [17]. This sample included all asthmatics (positive answer to the question “Have you ever had asthma?” at either survey) as well as a random sample of non-asthmatic controls. 248 participants were removed due to several reasons, including elevated levels of the inflammatory marker hs-CRP (>10 mg/L, N = 54), leading to a discovery arm of 1392 individuals (Figure S1). The discovery arm contained 548 (39.4%) self-declared asthmatics, whereas there were no self-declared asthmatics in the replication arm. Both the discovery and the replication sample were submitted to a first step of fine mapping of *SERPINA1* resulting in 5569 individuals from whom all the selected SNPs could be successfully determined. In a second step, a subsample with abnormally low AAT measurements additionally underwent *SERPINA1* exon sequencing.

#### Additional Studies

The populations are briefly described in Table S8.

### Phenotype Measurements

AAT serum levels in SAPALDIA were determined by latex-enhanced immunoturbidimetric assays (Roche Diagnostics, on a Roche Cobas Integra analyzer) with interassay coefficients of variation below 5% and lower detection rate of 0.21 g/L. Serum concentrations in Copenhagen were measured by immunoturbidimetric assays (Thermo Scientific, on a Thermo Scientific Konelab analyzer) with coefficients of variation below 5% and lower detection rate of 0.10 g/L.

Lung function was measured in all participating studies by spirometry without bronchodilation ([52] and Table S8). In the patient-based studies, in which a lung resection was carried out (Groningen, UBC), lung function measurements were carried out prior to the intervention.

### Genotyping

#### SAPALDIA

Genomic DNA was extracted from blood samples using the Puregene DNA Isolation Kit (Gentra Systems). Genotyping of the GWA-bound subset was performed on the Illumina Human 610quad array. Asthmatic and non-asthmatic samples were tested in random blinded order to avoid systematic array-related artifacts. 567,589 autosomal SNPs were satisfactorily genotyped (mean call rate: 99.7%). 69,892 were excluded from analysis due to violation of Hardy Weinberg Equilibrium (HWE, P<10^−4^), low call rate (<97%) or MAF<5%.

Genotyping of GWAS finding rs2566347 on chromosome 3 in the SAPALDIA replication arm was carried out using the MassARRAY iPLEX Gold (Sequenom).

The SNPs selected in the first fine mapping step were genotyped by polymerase chain reaction (PCR) with fluorescently labeled Taq-Man probes (Vic or Fam labels) on a Light Cycler 480 (Roche Diagnostics). All SNPs were in HWE (P>0.01) [20].

#### Additional Studies

PI S and Z genotypes were determined by PCR in Copenhagen and LHS as previously described [54], [55]. The SNP rs4905179 was genotyped in the course of GWAS projects in B58C, BHS, Copenhagen, Korcula, KORA S3, KORA F4, LBC36, LHS, NSPHS, ORCADES, Split, UBC, and Vis.

### Imputation

#### SAPALDIA

We have carried out genome-wide imputation from 60 CEU HapMap2 (release 22, NCBI build 36) reference panels [18] using MACH 1.0.16 [56] resulting in 2,588,592 autosomal HapMap-based SNPs. 2,168,668 SNPs fulfilled the quality criteria, which are as mentioned above for genotyped SNPs and additionally consisted of an imputation-r^2^>0.5.

Further imputation was carried out in the most promising loci using 566 EUR reference haplotypes from the August 2010 release of 1000G on the MACH (pre-phasing) and Minimac-omp programs. SNPs with an imputation-r^2^>0.5 and MAF>0.1% passed the quality check.

#### Additional Studies

Rs4905197 was imputed based on 1000G reference panels in Groningen, NFBC66, and SHIP (imputation-r^2^≥0.99). Rs3748312, rs17580 (PI S) and rs28929474 (PI Z) were 1000G imputed in B58C, BHS, Groningen, Korcula, KORA S3, KORA F4, LBC36, NFBC66, NSPHS, ORCADES, Split, SHIP, UBC, and Vis (imputation-r^2^ 0.86–0.98, 0.69–0.83, and 0.82–0.98, respectively).

### *SERPINA1* SNP Selection for Fine Mapping in SAPALDIA

In an attempt to find AAT modifying *SERPINA1* gene variants acting independently of each other, a multiple strategy to optimally cover the gene was applied. Sequencing of the whole *SERPINA1* gene in 25 unrelated samples from the Italian registry of AAT deficiency which demonstrated extreme phenotypes was used to identify common SNPs not present in HapMap. Extreme phenotypes consisted of 11 samples with AAT>1.60 g/L and hs-CRP <8 mg/L, 3 samples with PI ZZ or PI SZ genotype and AAT<0.20 g/L, 2 samples with PI MZ genotype and AAT<0.60 g/L, as well as 9 non-carriers of PI S or PI Z alleles with blood levels >0.65 and <1.10 g/L. In these 25 samples, a total of 129 mutations were identified in the *SERPINA1* gene. After removing SNPs which were monomorphic in our data, SNPs deviating from HWE or lying in high LD with an adjacent marker (D′>0.8 and r^2^>0.4 according to JLIN [57]), we finally obtained a list of 22 common SNPs (Table 4, selection A). In a second strategy, HapMap CEU data was used to select tagging SNPs (Haploview 3.32) [58], resulting in 8 polymorphisms (selection B). Third, TAMAL [59] was used to identify promising SNPs in the region of the *SERPINA1* gene (selection C). Pairwise LD and the feasibility of designing a corresponding TaqMan assay reduced the number of SNPs to 13. Two established (PI S and Z) and one suggestive (rs8004738 [60]) functional SNPs were added (selection D), resulting in 16 SNPs used in the conditional analysis. Three of them were already part of the SNP array genotyped for the GWAS. The 5 SNPs in coding regions (exons 2–5) were all non-synonymous.

### Exon Sequencing in SAPALDIA

We sequenced 410 individuals with abnormally low AAT levels with the Sanger chain-termination method. Different thresholds according to the deficiency genotypes and hs-CRP values were applied to define an abnormally low AAT concentration (PI MM: 1.13 g/L if hs-CRP >8 mg/L and 1.00 g/L if hs-CRP ≤8 mg/L; PI MS: 0.85 g/L; PI MZ: 0.65 g/L) [21]. The cut-off of 1.13 g/L was earlier reported to be the best to differentiate AAT-deficient patients from healthy individuals [61]. Since exon 1 is non-coding, the sequencing procedure was only applied to exons 2 to 5.

### Statistical Analysis

AAT serum levels were only marginally skewed to the right, and a log-transformation of these data was omitted since it led to a stronger deviation from normality. Student's t-test was used to compare adjusted mean AAT levels between different subgroups of the sequenced samples. The genome-wide association of 2.17 million quality-controlled SNPs with serum AAT levels was assessed using fixed effects linear regression with ProbABEL [62]. An additive genetic model was applied and the association was adjusted for sex, age, study center, dichotomous current smoking status, as well as population stratification factors. To account for population stratification, we relied on previously inferred ancestry-informative principal components using EIGENSTRAT 2.0 software [63] and HapMap data, as well as additional reference European samples [64]. Cryptic relatedness was detected based on identity-by-state (IBS) analysis. Influence of additional suggestive determinants of AAT, such as hs-CRP, BMI, alcohol intake and passive smoking was assessed in a sensitivity analysis. We also performed genome-wide analysis conditioned on the functionally established PI S and PI Z variants. Bonferroni correction for multiple testing was applied, resulting in P<5*10^−8^ to designate genome-wide significance, taking account of one million independent tests for common variants across the genome. For the SNPs imputed by using 1000G reference samples, we considered a three times lower p-value as adequate as roughly three times more SNPs on chromosome 14 passed an imputation-r^2^ threshold of 0.5 (219,471 1000G-derived variants vs. 82,296 HapMap2-derived variants). Applying this to a 2 Mb chromosomal stretch (with approximately 600 HapMap2-derived SNPs) resulted in a significance threshold of roughly 3*10^−5^.

For the replicated SNP in the SAPALDIA replication arm, as well as for the lung function analysis, a two-sided p-value of 0.05 was considered significant. We investigated heterogeneity between asthmatics and non-asthmatics in the discovery arm by testing for a difference between the two effects, using a chi-square test with one degree of freedom.

Replication analysis for AAT in Copenhagen, as well as association analyses of the 16 genotyped *SERPINA1* SNPs in both the SAPALDIA discovery and replication arm, was carried out applying the same statistical model as in the GWAS apart from the adjustment for population stratification factors. Stepwise conditional analyses were conducted by testing each SNP for AAT association after including at each step the most significantly associated SNP in the model. As some of these SNPs turned out to be in unexpectedly high LD, we applied a threshold level for statistical significance of P = 0.005, accounting for approximately ten independent tests [65].

To be as close as possible to the calculations carried out in the original publication [14], multivariate linear regression models for lung function analyses were used adjusted for sex, age, height and population stratification factors (if available).

All the SAPALDIA regression analyses were performed with STATA 12.1 IC.

### Further Software

Manhattan, Q-Q and forest plots were created with the help of R 2.15.1 (www.r-project.org). Regional association plots were drawn using LocusZoom [66]. Pairwise LD was calculated for HapMap2 and 1000G CEU data using SNAP [67]. The LD plot was produced with HaploView 4.2 [58]. The effect of non-synonymous SNPs on protein structure was predicted by SIFT [68]. Finally, Quanto 1.2.4 (hydra.usc.edu/gxe/) was used for power calculations for the GWAS.

## Supporting Information

Figure S1 SAPALDIA study design for the determination of AAT associated genetic variants.^a^ consisting of subjects with abnormally low AAT levels independent of PI S or Z alleles (see Materials and Methods).
(TIF)

Figure S2 Q-Q plot of genome-wide -log(10) p-values for association with AAT serum level.
(TIF)

Figure S3 Forest plot of meta-analyzed results for the effect per minor allele of rs17580 (PI S) on FEV1 in ever-smokers, adjusted for sex, age, height and population stratification factors. Studies based on population isolates with a high degree of cryptic relatedness are presented separately. Effect estimates of meta-analyses are shown with green diamonds. I^2^ is a measure of the heterogeneity between studies. Random effect meta-analyses are included if I^2^>0.5. Study weights (blue squares) correspond to the fixed effect meta-analyses.
(TIF)

Figure S4 Forest plot of meta-analyzed results for the effect per minor allele of rs28929474 (PI Z) on FEV1 in ever-smokers, adjusted for sex, age, height and population stratification factors. Studies based on population isolates with a high degree of cryptic relatedness are presented separately. Effect estimates of meta-analyses are shown with green diamonds. I^2^ is a measure of the heterogeneity between studies. Random effect meta-analyses are included if I^2^>0.5. Study weights (blue squares) correspond to the fixed effect meta-analyses.
(TIF)

Table S1 Characteristics of SAPALDIA follow-up participants belonging to the discovery (N = 1392) and replication arm (N = 4245), and of participants of the Copenhagen City Heart Study (N = 8273).
(DOC)

Table S2 Characteristics of study populations contributing to the SNP association analyses with FEV1.
(XLS)

Table S3 The top 100 ranking SNPs associated with AAT serum level in SAPALDIA (N = 1392).
(DOC)

Table S4 *SERPINA* regional variants based on 1000 Genomes imputation reaching statistical significance for the association with AAT serum level in SAPALDIA (N = 1392).
(DOC)

Table S5 The top 100 ranking SNPs associated with AAT serum level, conditional on PI S and Z alleles in SAPALDIA (N = 1392).
(DOC)

Table S6 Accuracy of 1000 Genomes based imputation in the *SERPINA1* region in SAPALDIA (N = 1392).
(DOC)

Table S7 Further variants in the *SERPINA1* coding region, present in a SAPALDIA subsample with abnormally low AAT serum levels (N = 410).
(DOC)

Table S8 Descriptions and acknowledgments of individual studies contributing to the SNP association analyses with FEV1.
(XLS)
