# Supplementary figures and images for: Causal and Synthetic Associations of Variants in the SERPINA Gene Cluster with Alpha1-antitrypsin Serum Levels
Source: PLoS Genet. 2013 Aug 22;9(8):e1003585. doi: 10.1371/journal.pgen.1003585 (PMC3749935; doi:10.1371/journal.pgen.1003585)

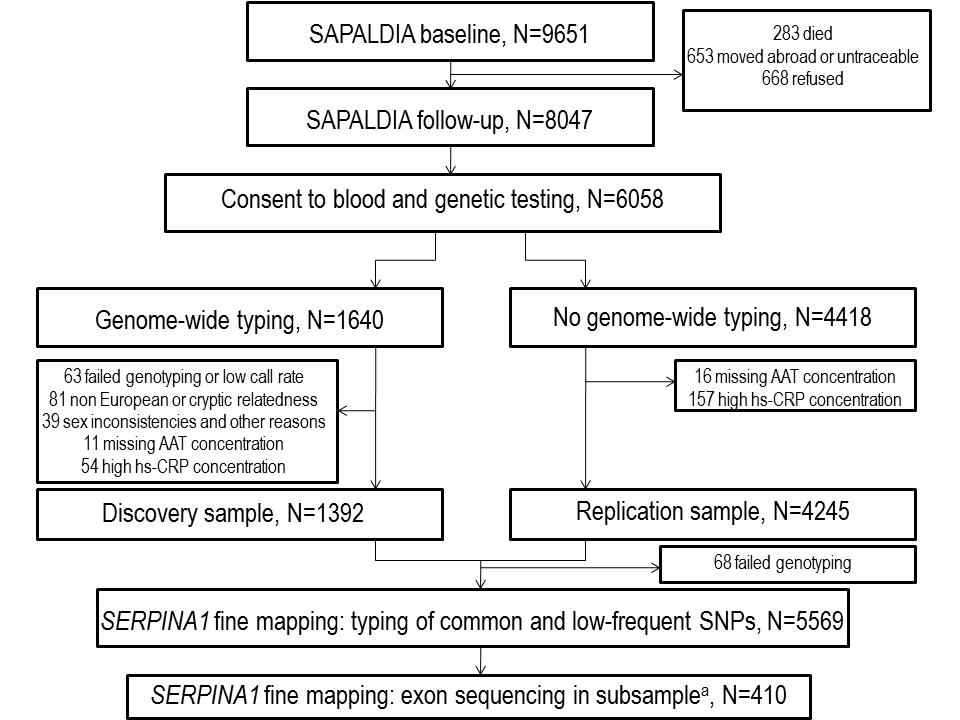

Supplement: Figure S1 — SAPALDIA study design for the determination of AAT associated genetic variants.a consisting of subjects with abnormally low AAT levels independent of PI S or Z alleles (see Materials and Methods). (TIF) [file pgen.1003585.s001.tif]

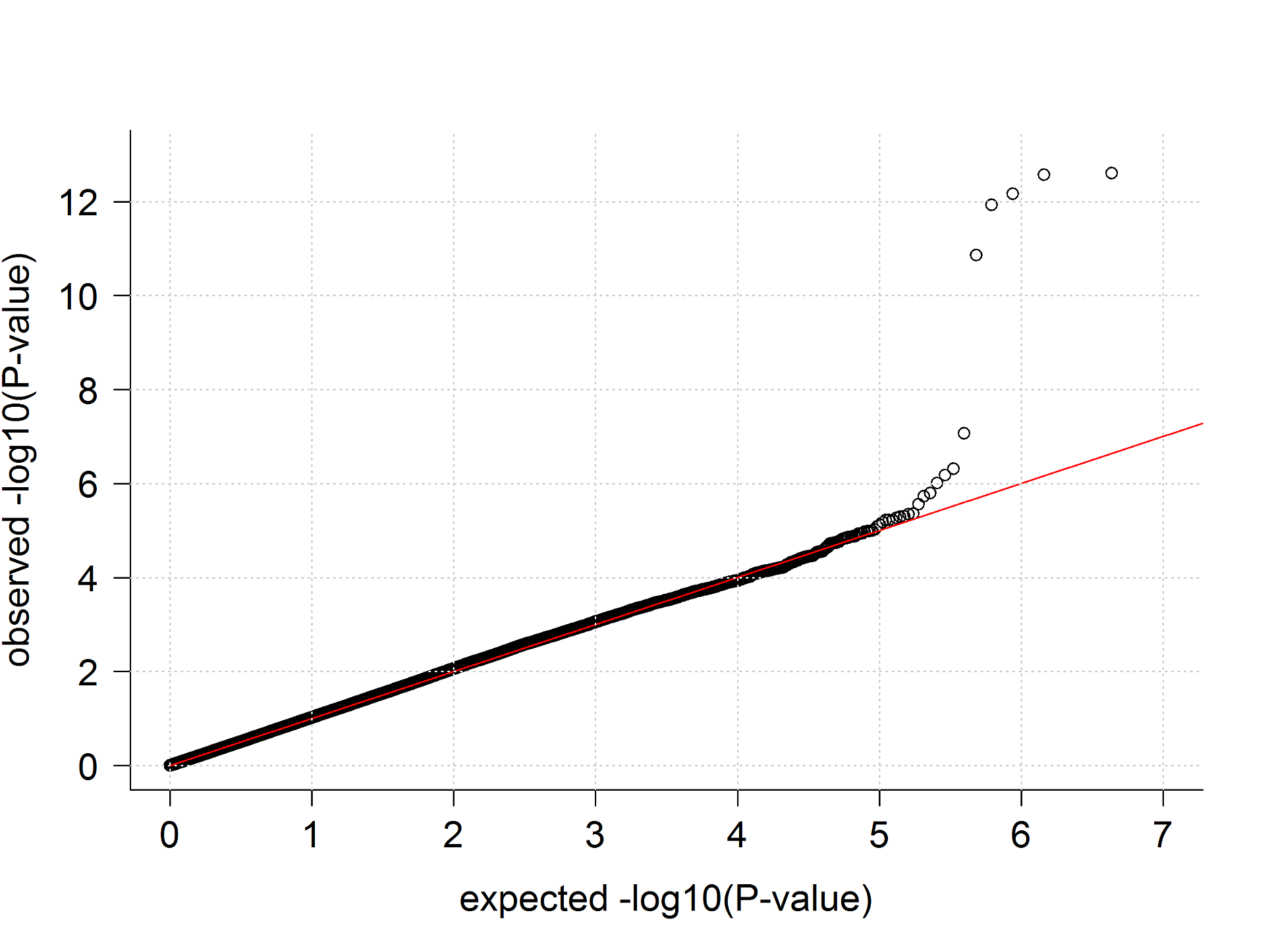

Supplement: Figure S2 — Q-Q plot of genome-wide -log(10) p-values for association with AAT serum level. (TIF) [file pgen.1003585.s002.tif]

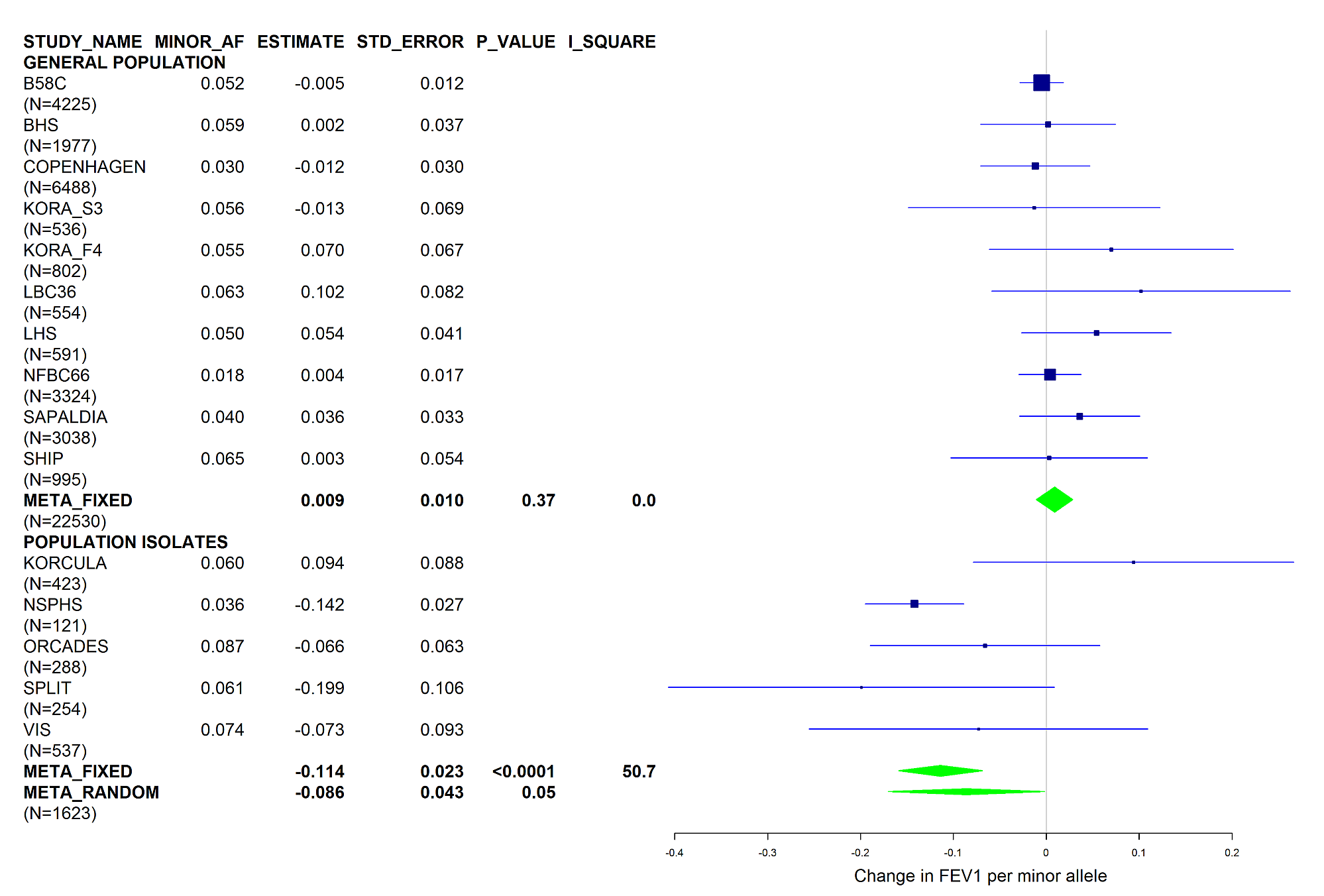

Supplement: Figure S3 — Forest plot of meta-analyzed results for the effect per minor allele of rs17580 (PI S) on FEV1 in ever-smokers, adjusted for sex, age, height and population stratification factors. Studies based on population isolates with a high degree of cryptic relatedness are presented separately. Effect estimates of meta-analyses are shown with green diamonds. I2 is a measure of the heterogeneity between studies. Random effect meta-analyses are included if I2>0.5. Study weights (blue squares) correspond to the fixed effect meta-analyses. (TIF) [file pgen.1003585.s003.tif]

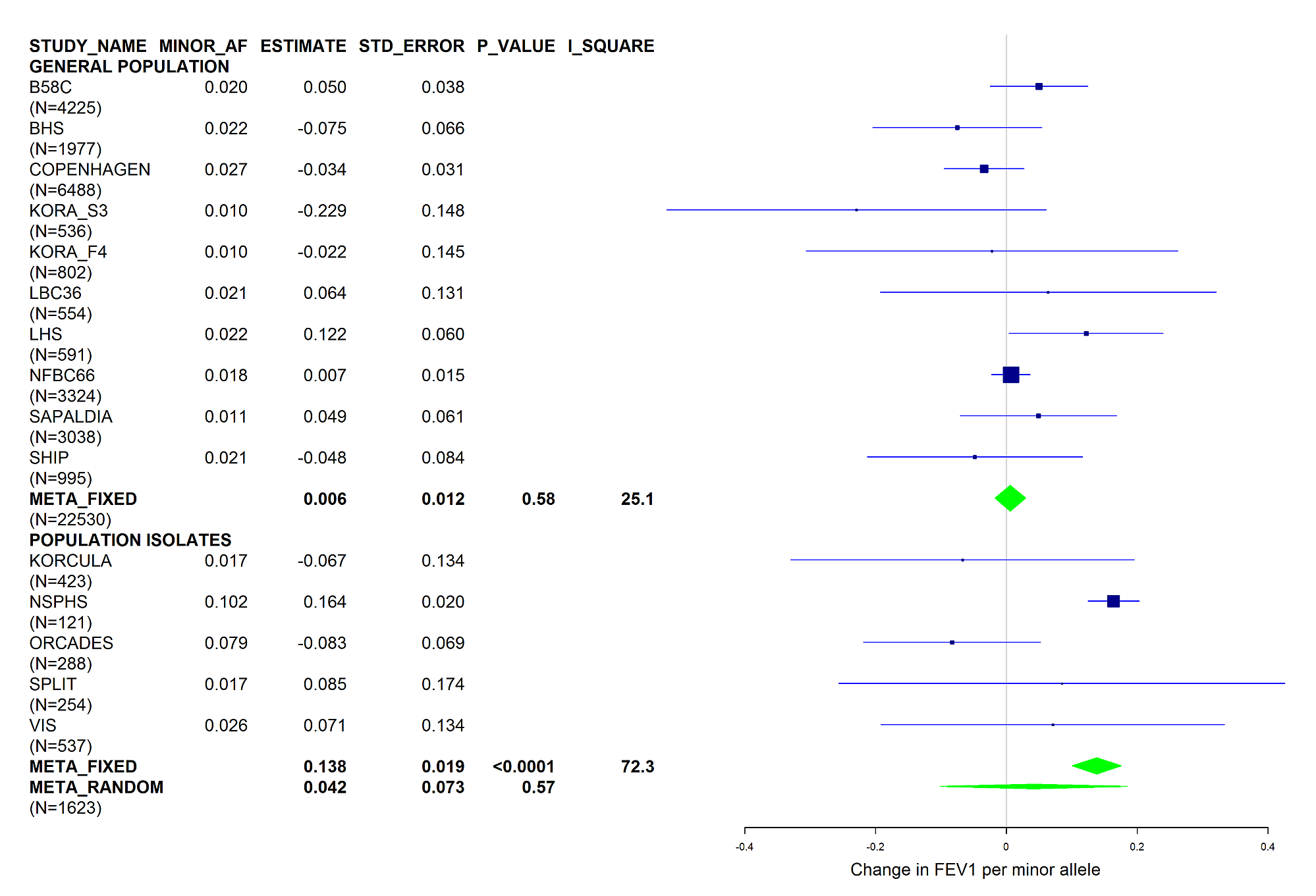

Supplement: Figure S4 — Forest plot of meta-analyzed results for the effect per minor allele of rs28929474 (PI Z) on FEV1 in ever-smokers, adjusted for sex, age, height and population stratification factors. Studies based on population isolates with a high degree of cryptic relatedness are presented separately. Effect estimates of meta-analyses are shown with green diamonds. I2 is a measure of the heterogeneity between studies. Random effect meta-analyses are included if I2>0.5. Study weights (blue squares) correspond to the fixed effect meta-analyses. (TIF) [file pgen.1003585.s004.tif]
