# Supplementary material for: Causal and Synthetic Associations of Variants in the SERPINA Gene Cluster with Alpha1-antitrypsin Serum Levels
Source: PLoS Genet. 2013 Aug 22;9(8):e1003585. doi: 10.1371/journal.pgen.1003585 (PMC3749935; doi:10.1371/journal.pgen.1003585)
Supplement: Table S1 — Characteristics of SAPALDIA follow-up participants belonging to the discovery (N = 1392) and replication arm (N = 4245), and of participants of the Copenhagen City Heart Study (N = 8273). (DOC) [file pgen.1003585.s005.doc]

Table S1. Characteristics of SAPALDIA follow-up participants belonging to the discovery (N=1392) and replication arm (N=4245), and of participants of the Copenhagen City Heart Study (N=8273).

|  | **SAPALDIA discovery** | **SAPALDIA replication** | **Copenhagen City Heart Study** |
| --- | --- | --- | --- |
| Age (mean; SD), years | 52.17; 11.18 | 52.17; 11.40 | 57.91; 15.12 |
| % women | 51.29 | 50.27 | 55.12 |
| % current smokers | 23.20 | 25.06 (N=4237) | 48.29 |
| % asthmatics^a^ | 39.37 | 0 (N=4239) | 6.01 |
| FEV1 (mean; SD), mL | 3530; 859 (N=1329) | 3607; 828 (N=4032) | 2728; 1021 |
| % airflow obstructive^b^ | 25.14 (N=1281) | 20.48 (N=3916) | 17.42 |
| AAT blood levels (mean; SD), g/L | 1.257; 0.200 | 1.255; 0.199 | 1.339; 0.276 |

Abbreviations: AAT, alpha1-antitrypsin; FEV1, forced expiratory volume in one second; FVC, forced vital capacity; SD, standard deviation.

^a^ Defined as self-reported at baseline or follow-up.

^b^ Defined as pre-bronchodilation FEV1/FVC ratio <0.7.
