# Supplementary material for: Causal and Synthetic Associations of Variants in the SERPINA Gene Cluster with Alpha1-antitrypsin Serum Levels
Source: PLoS Genet. 2013 Aug 22;9(8):e1003585. doi: 10.1371/journal.pgen.1003585 (PMC3749935; doi:10.1371/journal.pgen.1003585)
Supplement: Table S3 — The top 100 ranking SNPs associated with AAT serum level in SAPALDIA (N = 1392). (DOC) [file pgen.1003585.s007.doc]

Table S3. The top 100 ranking SNPs associated with AAT serum level in SAPALDIA (N=1392).

| **SNP** | **Chromosome** | **Position** | **Gene** | **Location** | **Determination** | **MAF** | **Imp-r^2^** | **Allele Effect** | **P** |
| --- | --- | --- | --- | --- | --- | --- | --- | --- | --- |
| rs2736887 | 14 | 93882733 |  | intergenic | imputed | 0.185 | 0.950 | 0.071 | 2.48E-13 |
| rs926144 | 14 | 93883155 |  | intergenic | imputed | 0.186 | 0.950 | 0.071 | 2.72E-13 |
| rs7151526 | 14 | 93933389 | *SERPINA1* | 5‘UTR | imputed | 0.065 | 0.769 | 0.116 | 6.78E-13 |
| rs4905179 | 14 | 93865245 | *SERPINA6* | 5‘UTR | genotyped | 0.180 | 1.000 | 0.068 | 1.20E-12 |
| rs11621961 | 14 | 93839229 | *SERPINA6* | 3‘UTR | genotyped | 0.355 | 0.945 | 0.052 | 1.37E-11 |
| rs17751837 | 14 | 93937997 | *SERPINA1* | 5‘UTR | genotyped | 0.097 | 0.995 | 0.063 | 8.56E-08 |
| rs1028580 | 14 | 93919635 | *SERPINA1* | intron | imputed | 0.154 | 0.979 | 0.051 | 4.87E-07 |
| rs8010121 | 14 | 93920367 | *SERPINA1* | intron | genotyped | 0.155 | 0.999 | 0.049 | 6.64E-07 |
| rs3748312 | 14 | 93924017 | *SERPINA1* | intron | imputed | 0.148 | 0.846 | 0.053 | 9.84E-07 |
| rs17752593 | 14 | 94007781 | *SERPINA9* | intron | genotyped | 0.129 | 0.997 | 0.053 | 1.59E-06 |
| rs2566347 | 3 | 159974071 |  | intergenic | imputed | 0.192 | 0.998 | 0.044 | 1.87E-06 |
| rs4703798 | 5 | 79448084 | *SERINC5* | intron | genotyped | 0.386 | 0.999 | 0.035 | 2.73E-06 |
| rs11160184 | 14 | 94007744 | *SERPINA9* | intron | imputed | 0.144 | 0.933 | 0.049 | 4.42E-06 |
| rs1430414 | 3 | 159987697 | *MFSD1* | 5‘UTR | imputed | 0.137 | 0.984 | 0.048 | 4.44E-06 |
| rs1560417 | 3 | 159972476 |  | intergenic | imputed | 0.200 | 0.998 | 0.041 | 5.06E-06 |
| rs1560418 | 3 | 159972335 |  | intergenic | genotyped | 0.200 | 1.000 | 0.041 | 5.07E-06 |
| rs6761989 | 3 | 159983253 |  | intergenic | imputed | 0.137 | 0.993 | 0.047 | 5.44E-06 |
| rs17643917 | 3 | 159968433 |  | intergenic | imputed | 0.137 | 1.000 | 0.047 | 5.99E-06 |
| rs17643860 | 3 | 159967954 |  | intergenic | imputed | 0.137 | 1.000 | 0.047 | 6.02E-06 |
| rs17700475 | 3 | 159967627 |  | intergenic | genotyped | 0.137 | 1.000 | 0.047 | 6.04E-06 |
| rs6580353 | 5 | 139390871 | *NRG2* | intron | genotyped | 0.248 | 0.948 | 0.039 | 6.63E-06 |
| rs6893779 | 5 | 129825798 |  | intergenic | imputed | 0.188 | 0.980 | 0.041 | 7.63E-06 |
| rs6595951 | 5 | 129874072 |  | intergenic | genotyped | 0.182 | 0.999 | 0.042 | 8.17E-06 |
| rs12089980 | 1 | 150698555 |  | intergenic | genotyped | 0.091 | 1.000 | 0.054 | 9.47E-06 |
| rs7731657 | 5 | 129971218 |  | intergenic | imputed | 0.170 | 0.942 | 0.044 | 1.01E-05 |
| rs4836515 | 5 | 129965471 |  | intergenic | imputed | 0.170 | 0.951 | 0.044 | 1.03E-05 |
| rs10069896 | 5 | 129938264 |  | intergenic | imputed | 0.171 | 0.971 | 0.043 | 1.04E-05 |
| rs10035791 | 5 | 129919660 |  | intergenic | genotyped | 0.172 | 1.000 | 0.042 | 1.05E-05 |
| rs10039487 | 5 | 129980823 |  | intergenic | imputed | 0.169 | 0.941 | 0.044 | 1.13E-05 |
| rs10276467 | 7 | 2701239 | *AMZ1* | intron | genotyped | 0.089 | 0.987 | 0.056 | 1.18E-05 |
| rs6874868 | 5 | 129825862 |  | intergenic | imputed | 0.185 | 0.989 | 0.041 | 1.18E-05 |
| rs12516876 | 5 | 129818959 |  | intergenic | imputed | 0.185 | 0.989 | 0.041 | 1.28E-05 |
| rs7714333 | 5 | 6370260 | *FLJ33360* | intron | genotyped | 0.459 | 0.972 | 0.032 | 1.32E-05 |
| rs16882595 | 8 | 112874500 |  | intergenic | imputed | 0.243 | 0.987 | 0.038 | 1.33E-05 |
| rs12479315 | 2 | 56960739 |  | intergenic | imputed | 0.491 | 0.963 | 0.032 | 1.38E-05 |
| rs2216407 | 2 | 56963650 |  | intergenic | imputed | 0.491 | 0.969 | 0.032 | 1.39E-05 |
| rs12081541 | 1 | 150707990 |  | intergenic | imputed | 0.091 | 0.904 | 0.056 | 1.43E-05 |
| rs16861952 | 3 | 150735514 | *WWTR1* | intron | imputed | 0.074 | 0.981 | 0.060 | 1.45E-05 |
| rs7141205 | 14 | 93838612 | *SERPINA6* | 3‘UTR | genotyped | 0.179 | 0.954 | 0.043 | 1.49E-05 |
| rs13181256 | 5 | 129806017 |  | intergenic | imputed | 0.184 | 0.980 | 0.041 | 1.53E-05 |
| rs4150667 | 11 | 18339487 | *GTF2H1* | intron | genotyped | 0.310 | 0.999 | 0.034 | 1.62E-05 |
| rs4263026 | 18 | 71438977 |  | intergenic | imputed | 0.198 | 0.983 | 0.039 | 1.72E-05 |
| rs3863076 | 3 | 159969394 |  | intergenic | genotyped | 0.145 | 1.000 | 0.044 | 1.73E-05 |
| rs12497958 | 3 | 160010142 | *MFSD1* | intron | imputed | 0.109 | 1.000 | 0.050 | 1.82E-05 |
| rs6441226 | 3 | 160008917 | *MFSD1* | intron | genotyped | 0.109 | 1.000 | 0.050 | 1.82E-05 |
| rs256435 | 5 | 79441982 | *SERINC5* | 3‘UTR | imputed | 0.438 | 0.789 | 0.035 | 1.84E-05 |
| rs7624420 | 3 | 159993368 | *MFSD1* | 5‘UTR | imputed | 0.102 | 0.990 | 0.052 | 1.85E-05 |
| rs13184933 | 5 | 129873782 |  | intergenic | imputed | 0.203 | 0.935 | 0.039 | 1.86E-05 |
| rs2041342 | 7 | 2696291 | *AMZ1* | intron | imputed | 0.093 | 0.924 | 0.055 | 1.94E-05 |
| rs9348171 | 6 | 167009010 | *RPS6KA2* | intron | imputed | 0.207 | 0.916 | 0.038 | 2.18E-05 |
| rs12462442 | 19 | 18094195 | *MAST3* | intron | imputed | 0.142 | 1.000 | 0.043 | 2.30E-05 |
| rs11086090 | 19 | 18093754 | *MAST3* | intron | genotyped | 0.142 | 1.000 | 0.043 | 2.30E-05 |
| rs756322 | 10 | 72414636 |  | intergenic | imputed | 0.089 | 0.680 | 0.065 | 2.51E-05 |
| rs6420430 | 16 | 80703651 | *HSD17B2* | 3‘UTR | imputed | 0.094 | 0.961 | 0.053 | 2.59E-05 |
| rs1251581 | 1 | 76198013 | *ASB17* | 5‘UTR | imputed | 0.377 | 0.956 | 0.032 | 2.78E-05 |
| rs10771459 | 12 | 29026497 |  | intergenic | imputed | 0.436 | 0.994 | 0.030 | 2.80E-05 |
| rs10936162 | 3 | 160018817 | *MFSD1* | intron | imputed | 0.109 | 0.973 | 0.050 | 2.83E-05 |
| rs2399702 | 15 | 95849200 |  | intergenic | imputed | 0.078 | 0.638 | 0.070 | 2.86E-05 |
| rs12611216 | 19 | 18095005 | *MAST3* | intron | imputed | 0.145 | 0.984 | 0.042 | 2.89E-05 |
| rs8104096 | 19 | 2561822 | *GNG7* | intron | genotyped | 0.253 | 0.979 | 0.035 | 2.89E-05 |
| rs1689271 | 1 | 76165450 | *ASB17* | intron | imputed | 0.365 | 0.948 | 0.032 | 3.05E-05 |
| rs12372252 | 12 | 29025493 |  | intergenic | imputed | 0.436 | 0.995 | 0.030 | 3.15E-05 |
| rs12605699 | 18 | 71433743 |  | intergenic | imputed | 0.190 | 0.857 | 0.041 | 3.23E-05 |
| rs17290460 | 13 | 73463470 | *KLF12* | intron | imputed | 0.096 | 0.927 | 0.052 | 3.35E-05 |
| rs2281143 | 6 | 167015841 | *RPS6KA2* | intron | imputed | 0.222 | 0.986 | 0.035 | 3.45E-05 |
| rs4835994 | 5 | 129749756 |  | intergenic | imputed | 0.173 | 0.972 | 0.040 | 3.46E-05 |
| rs1658737 | 1 | 76240227 |  | intergenic | imputed | 0.371 | 0.991 | 0.031 | 3.47E-05 |
| rs1251531 | 1 | 76241922 |  | intergenic | genotyped | 0.371 | 1.000 | 0.031 | 3.48E-05 |
| rs10484524 | 6 | 167016267 | *RPS6KA2* | intron | genotyped | 0.224 | 0.999 | 0.035 | 3.55E-05 |
| rs2623960 | 6 | 152935134 | *SYNE1* | intron | genotyped | 0.232 | 0.999 | 0.036 | 3.57E-05 |
| rs1251551 | 1 | 76236265 |  | intergenic | imputed | 0.370 | 0.985 | 0.031 | 3.58E-05 |
| rs1251550 | 1 | 76234909 |  | intergenic | imputed | 0.370 | 0.989 | 0.031 | 3.69E-05 |
| rs17218496 | 13 | 73468873 | *KLF12* | intron | imputed | 0.099 | 0.950 | 0.051 | 3.72E-05 |
| rs10145569 | 14 | 70722923 | *c14orf56* | 3‘UTR | imputed | 0.400 | 0.972 | 0.031 | 3.76E-05 |
| rs740692 | 19 | 18095567 | *MAST3* | intron | imputed | 0.147 | 0.980 | 0.041 | 3.81E-05 |
| rs6458184 | 6 | 40873668 |  | intergenic | imputed | 0.271 | 0.992 | 0.034 | 3.86E-05 |
| rs17290439 | 13 | 73462261 | *KLF12* | intron | imputed | 0.098 | 0.911 | 0.052 | 3.92E-05 |
| rs1796812 | 1 | 76174419 | *ASB17* | 5‘UTR | imputed | 0.356 | 0.989 | 0.031 | 3.94E-05 |
| rs4695888 | 4 | 174998288 |  | intergenic | genotyped | 0.500 | 0.998 | 0.029 | 3.97E-05 |
| rs10843288 | 12 | 29023614 |  | intergenic | imputed | 0.437 | 0.996 | 0.030 | 4.03E-05 |
| rs6930345 | 6 | 40873538 |  | intergenic | genotyped | 0.271 | 0.999 | 0.034 | 4.20E-05 |
| rs7299873 | 12 | 29029495 |  | intergenic | imputed | 0.440 | 0.989 | 0.030 | 4.28E-05 |
| rs1741547 | 4 | 3491737 | *LRPAP1* | intron | imputed | 0.300 | 0.949 | 0.033 | 4.32E-05 |
| rs6574008 | 14 | 70722644 | *c14orf56* | 3‘UTR | imputed | 0.402 | 0.964 | 0.031 | 4.39E-05 |
| rs10771458 | 12 | 29023602 |  | intergenic | imputed | 0.437 | 0.997 | 0.030 | 4.40E-05 |
| rs1794444 | 4 | 3487530 | *LRPAP1* | intron | imputed | 0.300 | 0.949 | 0.033 | 4.48E-05 |
| rs6564966 | 16 | 80700610 | *HSD17B2* | 3‘UTR | imputed | 0.082 | 0.906 | 0.056 | 4.65E-05 |
| rs2184658 | 1 | 219119080 | *HLX* | 5‘UTR | imputed | 0.179 | 0.936 | 0.039 | 4.66E-05 |
| rs11049978 | 12 | 29030367 |  | intergenic | imputed | 0.440 | 0.992 | 0.030 | 4.70E-05 |
| rs1850629 | 6 | 118200284 |  | intergenic | imputed | 0.092 | 0.744 | 0.059 | 4.73E-05 |
| rs11049971 | 12 | 29021467 |  | intergenic | genotyped | 0.438 | 0.999 | 0.030 | 4.80E-05 |
| rs740693 | 19 | 18095639 | *MAST3* | intron | imputed | 0.148 | 0.974 | 0.040 | 4.88E-05 |
| rs6789 | 4 | 3484493 | *LRPAP1* | 3‘UTR | imputed | 0.301 | 0.952 | 0.033 | 4.98E-05 |
| rs1359422 | 13 | 73480470 | *KLF12* | intron | genotyped | 0.098 | 0.999 | 0.049 | 5.03E-05 |
| rs1607369 | 6 | 118187203 | *NUS1* | 3‘UTR | imputed | 0.091 | 0.735 | 0.059 | 5.20E-05 |
| rs973229 | 5 | 129775011 |  | intergenic | imputed | 0.171 | 0.983 | 0.039 | 5.26E-05 |
| rs3738182 | 1 | 219124285 | *HLX* | exon | imputed | 0.178 | 0.933 | 0.039 | 5.40E-05 |
| rs7303799 | 12 | 29033635 |  | intergenic | imputed | 0.425 | 0.957 | 0.030 | 5.45E-05 |
| rs2189378 | 5 | 129763506 |  | intergenic | imputed | 0.169 | 0.980 | 0.039 | 5.46E-05 |
| rs1489078 | 12 | 29032680 |  | intergenic | genotyped | 0.441 | 1.000 | 0.029 | 5.77E-05 |

Abbreviations: AAT, alpha1-antitrypsin; MAF, minor allele frequency; SNP, single nucleotide polymorphism.

Imp-r^2^ is an indicator of imputation quality. SNPs with MAF <0.05 or imp-r^2^ <0.5 were excluded.

Chromosomal position is based on reference panel, NCBI build 36.3. Allele Effects are shown in absolute numbers.
