# Supplementary material for: Causal and Synthetic Associations of Variants in the SERPINA Gene Cluster with Alpha1-antitrypsin Serum Levels
Source: PLoS Genet. 2013 Aug 22;9(8):e1003585. doi: 10.1371/journal.pgen.1003585 (PMC3749935; doi:10.1371/journal.pgen.1003585)
Supplement: Table S4 — SERPINA regional variants based on 1000 Genomes imputation reaching statistical significance for the association with AAT serum level in SAPALDIA (N = 1392). (DOC) [file pgen.1003585.s008.doc]

Table S4. *SERPINA* regional variants based on 1000 Genomes imputation reaching statistical significance for the association with AAT serum level in SAPALDIA (N=1392).

| **SNP** | **Position** | **Gene** | **Location** | **MAF** | **Imp-r^2^** | **Allele Effect** | **P** | **GWAS inclusion** |
| --- | --- | --- | --- | --- | --- | --- | --- | --- |
| rs28929474 | 94844947 | *SERPINA1* | exon | 0.008 | 0.684 | 0.620 | 4.61E-43 | no |
| rs112458284 | 94672731 | *PPP4R4* | intron | 0.028 | 0.578 | 0.363 | 5.35E-32 | no |
| rs149837463 | 94632757 |  | intergenic | 0.022 | 0.529 | 0.388 | 1.09E-26 | no |
| rs111974986 | 94533735 | *DDX24* | intron | 0.017 | 0.641 | 0.332 | 8.33E-20 | no |
| rs7151526 | 94863636 | *SERPINA1* | 5‘UTR | 0.057 | 0.702 | 0.144 | 5.88E-15 | yes |
| rs61980636 | 94784618 | *SERPINA6* | intron | 0.181 | 0.977 | 0.070 | 2.70E-13 | no, but in high LD |
| rs2736887 | 94812980 |  | intergenic | 0.195 | 0.980 | 0.068 | 4.91E-13 | yes |
| rs926144 | 94813402 |  | intergenic | 0.195 | 0.980 | 0.068 | 5.05E-13 | yes |
| rs965344 | 94818078 |  | intergenic | 0.195 | 0.980 | 0.068 | 6.62E-13 | no, but in high LD |
| rs4905179 | 94795492 | *SERPINA6* | 5‘UTR | 0.184 | 1.000 | 0.068 | 6.90E-13 | yes |
| rs61280460 | 94796184 | *SERPINA6* | 5‘UTR | 0.184 | 1.000 | 0.068 | 6.98E-13 | no, but in high LD |
| rs7149605 | 94854041 | *SERPINA1* | intron | 0.095 | 0.853 | 0.084 | 5.14E-11 | no, but in high LD |
| rs11621961 | 94769476 | *SERPINA6* | 3‘UTR | 0.353 | 0.986 | 0.048 | 9.52E-11 | yes |
| rs75416602 | 94554409 | *IFI27L1* | intron | 0.041 | 0.897 | 0.122 | 3.12E-09 | no |
| rs79811936 | 94510275 | *OTUB2* | intron | 0.040 | 0.824 | 0.122 | 1.04E-08 | no, but in high LD |
| rs74712407 | 94506729 | *OTUB2* | intron | 0.040 | 0.823 | 0.122 | 1.16E-08 | no, but in high LD |
| rs55862705 | 94866482 | *SERPINA1* | 5‘UTR | 0.093 | 0.949 | 0.070 | 1.49E-08 | no, but in high LD |
| rs17751837 | 94868244 | *SERPINA1* | 5‘UTR | 0.099 | 0.998 | 0.063 | 6.54E-08 | yes |
| rs145730801 | 94768196 | *SERPINA6* | 3‘UTR | 0.052 | 0.608 | 0.112 | 7.01E-08 | no |
| rs35306951 | 94873695 |  | intergenic | 0.098 | 0.971 | 0.064 | 9.35E-08 | no, but in high LD |
| rs8015929 | 94763787 |  | intergenic | 0.370 | 0.635 | 0.048 | 1.78E-07 | no |
| rs3748312 | 94854264 | *SERPINA1* | intron | 0.161 | 0.864 | 0.055 | 2.09E-07 | yes |
| rs7146221 | 94769081 | *SERPINA6* | 3‘UTR | 0.443 | 0.902 | 0.039 | 3.57E-07 | no |
| rs72692809 | 94904542 | *SERPINA11* | 3‘UTR | 0.104 | 0.583 | 0.076 | 3.64E-07 | no |
| rs55683719 | 94936222 | *SERPINA9* | intron | 0.136 | 0.791 | 0.058 | 4.42E-07 | no, but in high LD |
| rs1028580 | 94849882 | *SERPINA1* | intron | 0.163 | 0.998 | 0.050 | 5.35E-07 | yes |
| rs8010121 | 94850614 | *SERPINA1* | intron | 0.163 | 1.000 | 0.049 | 5.90E-07 | yes |
| rs72704312 | 94733695 | *PPP4R4* | intron | 0.072 | 0.541 | 0.092 | 9.09E-07 | no |
| rs61976073 | 95050970 | *SERPINA5* | intron | 0.075 | 0.861 | 0.067 | 9.95E-07 | no |
| rs17752593 | 94938028 | *SERPINA9* | intron | 0.123 | 0.981 | 0.053 | 1.59E-06 | yes |
| rs11160167 | 94763621 |  | intergenic | 0.495 | 0.748 | 0.039 | 2.29E-06 | no |
| rs56149519 | 94554061 | *IFI27L1* | intron | 0.067 | 0.803 | 0.077 | 2.34E-06 | no, but in high LD |
| rs11160184 | 94937991 | *SERPINA9* | intron | 0.150 | 0.903 | 0.048 | 5.29E-06 | yes |
| rs11622665 | 94774995 | *SERPINA6* | intron | 0.160 | 0.988 | 0.045 | 5.98E-06 | yes |
| rs12892767 | 94767211 | *SERPINA6* | 3‘UTR | 0.444 | 0.831 | 0.035 | 9.58E-06 | no |
| rs61976081 | 95066611 |  | intergenic | 0.084 | 0.693 | 0.064 | 1.55E-05 | no |

Abbreviations: AAT, alpha1-antitrypsin; LD, linkage disequilibrium; MAF, minor allele frequency; SNP, single nucleotide polymorphism.

Imp-r^2^ is an indicator of imputation quality. SNPs with MAF <0.001 or imp-r^2^ <0.5 were excluded. Presented are results with P<3*10(-5) within a 2 Mb region from 93,857 kb to 95,857 kb on chromosome 14.

Chromosomal position is based on panel GRCh37.p5, NCBI build 37.3. Allele Effects are shown in absolute numbers. High LD is defined as a LD-r^2^ >0.8 using SNAP software.
