# Supplementary material for: Causal and Synthetic Associations of Variants in the SERPINA Gene Cluster with Alpha1-antitrypsin Serum Levels
Source: PLoS Genet. 2013 Aug 22;9(8):e1003585. doi: 10.1371/journal.pgen.1003585 (PMC3749935; doi:10.1371/journal.pgen.1003585)
Supplement: Table S5 — The top 100 ranking SNPs associated with AAT serum level, conditional on PI S and Z alleles in SAPALDIA (N = 1392). (DOC) [file pgen.1003585.s009.doc]

Table S5. The top 100 ranking SNPs associated with AAT serum level, conditional on PI S and Z alleles in SAPALDIA (N=1392).

| **SNP** | **Chromosome** | **Position** | **Gene** | **Location** | **Determination** | **MAF** | **Imp-r^2^** | **Allele Effect** | **P** |
| --- | --- | --- | --- | --- | --- | --- | --- | --- | --- |
| rs2566347 | 3 | 159974071 |  | intergenic | imputed | 0.192 | 0.998 | 0.043 | 7.88E-08 |
| rs1560417 | 3 | 159972476 |  | intergenic | imputed | 0.200 | 0.998 | 0.042 | 1.11E-07 |
| rs1560418 | 3 | 159972335 |  | intergenic | genotyped | 0.200 | 1.000 | 0.042 | 1.11E-07 |
| rs1430414 | 3 | 159987697 | *MFSD1* | 5‘UTR | imputed | 0.137 | 0.984 | 0.045 | 9.26E-07 |
| rs6761989 | 3 | 159983253 |  | intergenic | imputed | 0.137 | 0.993 | 0.044 | 1.14E-06 |
| rs17643917 | 3 | 159968433 |  | intergenic | imputed | 0.137 | 1.000 | 0.044 | 1.23E-06 |
| rs17643860 | 3 | 159967954 |  | intergenic | imputed | 0.137 | 1.000 | 0.044 | 1.24E-06 |
| rs17700475 | 3 | 159967627 |  | intergenic | genotyped | 0.137 | 1.000 | 0.044 | 1.25E-06 |
| rs3863076 | 3 | 159969394 |  | intergenic | genotyped | 0.145 | 1.000 | 0.042 | 1.69E-06 |
| rs2206593 | 1 | 184909052 | *PTGS2* | 3‘UTR | genotyped | 0.065 | 0.956 | 0.060 | 4.60E-06 |
| rs12462442 | 19 | 18094195 | *MAST3* | intron | imputed | 0.142 | 1.000 | 0.040 | 5.74E-06 |
| rs11086090 | 19 | 18093754 | *MAST3* | intron | genotyped | 0.142 | 1.000 | 0.040 | 5.74E-06 |
| rs9541793 | 13 | 68628588 |  | intergenic | imputed | 0.258 | 0.990 | 0.032 | 7.37E-06 |
| rs1446378 | 13 | 68626607 |  | intergenic | imputed | 0.258 | 0.994 | 0.032 | 7.45E-06 |
| rs4703798 | 5 | 79448084 | *SERINC5* | intron | genotyped | 0.386 | 0.999 | 0.029 | 7.50E-06 |
| rs1446379 | 13 | 68626323 |  | intergenic | imputed | 0.258 | 0.995 | 0.032 | 7.61E-06 |
| rs13400830 | 2 | 12972015 |  | intergenic | genotyped | 0.083 | 0.989 | 0.051 | 7.83E-06 |
| rs1023818 | 13 | 68625043 |  | intergenic | genotyped | 0.258 | 1.000 | 0.031 | 8.04E-06 |
| rs16861952 | 3 | 150735514 | *WWTR1* | intron | imputed | 0.074 | 0.981 | 0.054 | 8.28E-06 |
| rs16859504 | 2 | 12972809 |  | intergenic | imputed | 0.081 | 0.926 | 0.053 | 8.38E-06 |
| rs12611216 | 19 | 18095005 | *MAST3* | intron | imputed | 0.145 | 0.984 | 0.039 | 1.01E-05 |
| rs6682093 | 1 | 179309512 | *MR1* | 3‘UTR | imputed | 0.362 | 0.983 | 0.029 | 1.03E-05 |
| rs1119065 | 1 | 184924695 | *PTGS2* | 5‘UTR | imputed | 0.066 | 0.810 | 0.062 | 1.03E-05 |
| rs1013665 | 1 | 184814763 |  | intergenic | imputed | 0.177 | 0.809 | 0.040 | 1.05E-05 |
| rs7514491 | 1 | 184865641 |  | intergenic | genotyped | 0.173 | 0.998 | 0.036 | 1.19E-05 |
| rs9599423 | 13 | 68623359 |  | intergenic | imputed | 0.261 | 0.989 | 0.031 | 1.31E-05 |
| rs6662507 | 1 | 179322783 | *IER5* | 5‘UTR | imputed | 0.346 | 0.955 | 0.029 | 1.42E-05 |
| rs9511045 | 13 | 23564937 | *SPATA13* | 5‘UTR | imputed | 0.112 | 0.931 | 0.044 | 1.44E-05 |
| rs7300338 | 12 | 4010161 | *LOC100507511* | 3‘UTR | imputed | 0.384 | 0.865 | 0.030 | 1.61E-05 |
| rs9541789 | 13 | 68620213 |  | intergenic | imputed | 0.262 | 0.985 | 0.030 | 1.63E-05 |
| rs3856068 | 1 | 179312991 | *MR1* | 3‘UTR | imputed | 0.365 | 0.982 | 0.028 | 1.67E-05 |
| rs9529519 | 13 | 68620322 |  | intergenic | imputed | 0.262 | 0.986 | 0.030 | 1.68E-05 |
| rs740692 | 19 | 18095567 | *MAST3* | intron | imputed | 0.147 | 0.980 | 0.037 | 1.74E-05 |
| rs2275470 | 1 | 179332430 | *IER5* | 3‘UTR | imputed | 0.344 | 0.924 | 0.030 | 1.79E-05 |
| rs12479315 | 2 | 56960739 |  | intergenic | imputed | 0.491 | 0.963 | 0.027 | 1.86E-05 |
| rs2216407 | 2 | 56963650 |  | intergenic | imputed | 0.491 | 0.969 | 0.027 | 1.92E-05 |
| rs2304408 | 3 | 159803574 | *MLF1* | intron | imputed | 0.229 | 0.972 | 0.032 | 1.99E-05 |
| rs7624771 | 3 | 159818103 | *MLF1* | 3‘UTR | imputed | 0.228 | 0.982 | 0.032 | 2.28E-05 |
| rs11923661 | 3 | 71284796 | *FOXP1* | intron | genotyped | 0.446 | 0.999 | 0.027 | 2.34E-05 |
| rs453176 | 21 | 45407444 | *ADARB1* | intron | imputed | 0.053 | 0.932 | 0.063 | 2.37E-05 |
| rs407550 | 21 | 45408504 | *ADARB1* | intron | imputed | 0.053 | 0.932 | 0.063 | 2.37E-05 |
| rs17699324 | 3 | 159907255 | *RARRES1* | intron | genotyped | 0.225 | 0.998 | 0.032 | 2.40E-05 |
| rs17699103 | 3 | 159902483 | *RARRES1* | intron | imputed | 0.225 | 0.976 | 0.032 | 2.41E-05 |
| rs7610009 | 3 | 159903063 | *RARRES1* | intron | imputed | 0.225 | 0.978 | 0.032 | 2.41E-05 |
| rs2745557 | 1 | 184915844 | *PTGS2* | intron | imputed | 0.179 | 0.882 | 0.037 | 2.43E-05 |
| rs3867391 | 3 | 159898534 | *RARRES1* | intron | imputed | 0.226 | 0.970 | 0.032 | 2.44E-05 |
| rs17698754 | 3 | 159894398 | *GFM1* | 3‘UTR | imputed | 0.226 | 0.968 | 0.032 | 2.46E-05 |
| rs9541803 | 13 | 68639250 |  | intergenic | imputed | 0.280 | 0.988 | 0.029 | 2.46E-05 |
| rs17004749 | 21 | 45418411 | *ADARB1* | intron | imputed | 0.052 | 0.941 | 0.063 | 2.54E-05 |
| rs6776901 | 3 | 159843286 | *GFM1* | 5‘UTR | imputed | 0.228 | 0.991 | 0.032 | 2.55E-05 |
| rs6777231 | 3 | 159843638 | *GFM1* | 5‘UTR | imputed | 0.228 | 0.989 | 0.032 | 2.56E-05 |
| rs17698494 | 3 | 159888567 | *GFM1* | intron | imputed | 0.227 | 0.969 | 0.032 | 2.57E-05 |
| rs740693 | 19 | 18095639 | *MAST3* | intron | imputed | 0.148 | 0.974 | 0.037 | 2.59E-05 |
| rs17697458 | 3 | 159859048 | *GFM1* | intron | imputed | 0.227 | 0.970 | 0.032 | 2.62E-05 |
| rs1522178 | 3 | 71286953 | *FOXP1* | intron | imputed | 0.449 | 0.972 | 0.027 | 2.63E-05 |
| rs7279483 | 21 | 45421702 | *ADARB1* | intron | imputed | 0.052 | 0.945 | 0.063 | 2.65E-05 |
| rs17630607 | 3 | 159872554 | *GFM1* | intron | imputed | 0.227 | 0.974 | 0.032 | 2.67E-05 |
| rs4338667 | 13 | 67094362 |  | intergenic | genotyped | 0.184 | 0.998 | 0.034 | 2.71E-05 |
| rs6971526 | 7 | 66998462 |  | intergenic | genotyped | 0.479 | 0.999 | 0.026 | 2.74E-05 |
| rs480075 | 1 | 184722959 | *PDC* | 5‘UTR | imputed | 0.179 | 0.693 | 0.041 | 2.76E-05 |
| rs9390650 | 6 | 149363751 | *UST* | intron | genotyped | 0.409 | 1.000 | 0.027 | 2.78E-05 |
| rs9541790 | 13 | 68621026 |  | intergenic | genotyped | 0.283 | 0.999 | 0.028 | 2.90E-05 |
| rs497285 | 1 | 184721052 | *PDC* | 5‘UTR | imputed | 0.180 | 0.693 | 0.041 | 2.94E-05 |
| rs2745559 | 1 | 184918625 | *PTGS2* | 5‘UTR | imputed | 0.179 | 0.862 | 0.037 | 3.03E-05 |
| rs1983870 | 13 | 23568163 | *SPATA13* | 5‘UTR | genotyped | 0.105 | 0.992 | 0.043 | 3.04E-05 |
| rs7770997 | 6 | 149373032 | *UST* | intron | imputed | 0.411 | 0.987 | 0.027 | 3.16E-05 |
| rs10172921 | 2 | 12960321 |  | intergenic | imputed | 0.093 | 0.956 | 0.045 | 3.30E-05 |
| rs9484838 | 6 | 144385865 | *PLAGL1* | intron | imputed | 0.132 | 0.942 | 0.039 | 3.34E-05 |
| rs10182783 | 2 | 12939134 |  | intergenic | imputed | 0.124 | 0.837 | 0.043 | 3.37E-05 |
| rs4835994 | 5 | 129749756 |  | intergenic | imputed | 0.173 | 0.972 | 0.035 | 3.38E-05 |
| rs7653672 | 3 | 71284144 | *FOXP1* | intron | imputed | 0.448 | 0.963 | 0.027 | 3.45E-05 |
| rs4263026 | 18 | 71438977 |  | intergenic | imputed | 0.198 | 0.983 | 0.033 | 3.47E-05 |
| rs11099924 | 4 | 155070285 |  | intergenic | imputed | 0.405 | 0.920 | 0.028 | 3.50E-05 |
| rs13388631 | 2 | 12960274 |  | intergenic | imputed | 0.093 | 0.961 | 0.045 | 3.56E-05 |
| rs11593082 | 10 | 8080437 | *TAF3* | intron | genotyped | 0.077 | 0.997 | 0.048 | 3.69E-05 |
| rs447590 | 13 | 112495212 | *ATP11A* | intron | imputed | 0.427 | 0.902 | 0.028 | 3.75E-05 |
| rs13395897 | 2 | 77520241 | *LRRTM4* | intron | imputed | 0.078 | 0.689 | 0.057 | 3.76E-05 |
| rs13430556 | 2 | 12960160 |  | intergenic | imputed | 0.093 | 0.968 | 0.045 | 3.82E-05 |
| rs4411966 | 4 | 155074041 |  | intergenic | imputed | 0.404 | 0.917 | 0.028 | 3.90E-05 |
| rs2688352 | 8 | 3816166 | *CSMD1* | intron | genotyped | 0.188 | 0.993 | 0.033 | 3.93E-05 |
| rs12489730 | 3 | 159940608 | *RARRES1* | 5‘UTR | imputed | 0.218 | 0.983 | 0.031 | 3.94E-05 |
| rs2460535 | 10 | 43141386 | *RASGEF1A* | 5‘UTR | imputed | 0.101 | 0.899 | 0.047 | 3.95E-05 |
| rs2399702 | 15 | 95849200 |  | intergenic | imputed | 0.078 | 0.638 | 0.060 | 3.95E-05 |
| rs4798376 | 18 | 5594240 | *EPB41L3* | 5‘UTR | genotyped | 0.254 | 0.992 | 0.030 | 4.03E-05 |
| rs10145569 | 14 | 70722923 | *c14orf56* | 3‘UTR | imputed | 0.400 | 0.972 | 0.027 | 4.03E-05 |
| rs13279316 | 8 | 112676764 |  | intergenic | genotyped | 0.260 | 0.998 | 0.029 | 4.09E-05 |
| rs12609740 | 19 | 18101298 | *MAST3* | intron | imputed | 0.149 | 0.968 | 0.036 | 4.16E-05 |
| rs13390247 | 2 | 12963014 |  | intergenic | imputed | 0.090 | 0.961 | 0.045 | 4.20E-05 |
| rs12698626 | 7 | 66991554 |  | intergenic | imputed | 0.476 | 0.915 | 0.026 | 4.20E-05 |
| rs2129818 | 12 | 16834202 |  | intergenic | genotyped | 0.399 | 1.000 | 0.026 | 4.28E-05 |
| rs7624420 | 3 | 159993368 | *MFSD1* | 5‘UTR | imputed | 0.102 | 0.990 | 0.043 | 4.43E-05 |
| rs6574008 | 14 | 70722644 | *c14orf56* | 3‘UTR | imputed | 0.402 | 0.964 | 0.027 | 4.51E-05 |
| rs1468488 | 22 | 15970744 | *IL17RA* | 3‘UTR | imputed | 0.291 | 0.922 | 0.029 | 4.52E-05 |
| rs6728142 | 2 | 12958541 |  | intergenic | imputed | 0.092 | 0.982 | 0.044 | 4.54E-05 |
| rs6930963 | 6 | 149396494 | *UST* | intron | imputed | 0.410 | 0.954 | 0.027 | 4.55E-05 |
| rs6893779 | 5 | 129825798 |  | intergenic | imputed | 0.188 | 0.980 | 0.033 | 4.63E-05 |
| rs8002029 | 13 | 23564535 | *SPATA13* | 5‘UTR | genotyped | 0.081 | 0.998 | 0.046 | 4.65E-05 |
| rs4622983 | 4 | 155074124 |  | intergenic | imputed | 0.405 | 0.910 | 0.027 | 4.71E-05 |
| rs1386819 | 12 | 16847868 |  | intergenic | imputed | 0.398 | 0.967 | 0.026 | 4.72E-05 |
| rs7984719 | 13 | 23564571 | *SPATA13* | 5‘UTR | imputed | 0.081 | 0.989 | 0.046 | 4.73E-05 |

Abbreviations: AAT, alpha1-antitrypsin; MAF, minor allele frequency; SNP, single nucleotide polymorphism.

Imp-r^2^ is an indicator of imputation quality. SNPs with MAF <0.05 or imp-r^2^ <0.5 were excluded.

Chromosomal position is based on reference panel, NCBI build 36.3. Allele Effects are shown in absolute numbers.
