# Supplementary material for: Causal and Synthetic Associations of Variants in the SERPINA Gene Cluster with Alpha1-antitrypsin Serum Levels
Source: PLoS Genet. 2013 Aug 22;9(8):e1003585. doi: 10.1371/journal.pgen.1003585 (PMC3749935; doi:10.1371/journal.pgen.1003585)
Supplement: Table S6 — Accuracy of 1000 Genomes based imputation in the SERPINA1 region in SAPALDIA (N = 1392). (DOC) [file pgen.1003585.s010.doc]

Table S6. Accuracy of 1000 Genomes based imputation in the *SERPINA1* region in SAPALDIA (N=1392).

| **SNP** | **Location** | **MAF (genotyped)** | **MAF (imputed)** |
| --- | --- | --- | --- |
| rs2896268 | 5’UTR | 0.491 | 0.488 |
| rs1956707 | 5’UTR | 0.036 | 0.037 |
| rs8004738 | exon 1 | 0.489 | 0.478 |
| rs1570142 | intron 1 | 0.488 | 0.478 |
| rs3748312 | intron 1 | 0.158 | 0.161 |
| rs3748316 | intron 1 | 0.178 | genotyped, Illumina 610quad |
| rs3748317 | intron 1 | 0.162 | 0.159 |
| rs1980617 | intron 1 | 0.393 | 0.385 |
| rs1980618 | intron 1 | 0.388 | 0.385 |
| rs2753935 | intron 1 | 0.428 | 0.440 |
| rs2144831 | intron 1 | 0.238 | 0.238 |
| rs709932 | exon 2 | 0.174 | genotyped, Illumina 610quad |
| rs6647 | exon 3 | 0.208 | 0.220 |
| rs17580 (S) | exon 3 | 0.040 | 0.057 |
| rs28929474 (Z) | exon 5 | 0.013 | 0.008 |
| rs1303 | exon 5 | 0.258 | genotyped, Illumina 610quad |

Abbreviations: GWAS, genome-wide association study; MAF, minor allele frequency; SNP, single nucleotide polymorphism.
