# Supplementary material for: Causal and Synthetic Associations of Variants in the SERPINA Gene Cluster with Alpha1-antitrypsin Serum Levels
Source: PLoS Genet. 2013 Aug 22;9(8):e1003585. doi: 10.1371/journal.pgen.1003585 (PMC3749935; doi:10.1371/journal.pgen.1003585)
Supplement: Table S7 — Further variants in the SERPINA1 coding region, present in a SAPALDIA subsample with abnormally low AAT serum levels (N = 410). (DOC) [file pgen.1003585.s011.doc]

Table S7. Further variants in the *SERPINA1* coding region, present in a SAPALDIA subsample with abnormally low AAT serum levels (N=410).

| **Variant, name** | **N**^a^ | **Location** | **Position** | **Function** | **Description** | **Computational prediction [68]** |
| --- | --- | --- | --- | --- | --- | --- |
| rs28931570, I | 9 | exon 2 | 93919141 | SNP, non-synonymous | deficiency variant [24] | damaging |
| ΔF52, Mmalton | 3 | exon 2 | 93919100-02 | codon deletion | deficiency variant [24] | n/a |
| rs111850950, M6passau | 5 | exon 2 | 93919078 | SNP, non-synonymous | neutral variant [27] | damaging |
| Q105P | 1 | exon 2 | 93918942 | SNP, non-synonymous | putative deficiency variant [21] | damaging |
| rs20546, M3riedenburg | 17 | exon 2 | 93918904 | SNP, synonymous | neutral variant [27] | n/a |
| rs112030253, V | 1 | exon 2 | 93918814 | SNP, non-synonymous | neutral variant [27] | tolerated |
| A153A | 1 | exon 2 | 93918797 | SNP, synonymous | novel | n/a |
| ΔY160, Q0granitefalls | 1 | exon 2 | 93918776 | nucleotide deletion | null variant [23] | n/a |
| rs28929470, F | 3 | exon 3 | 93917139 | SNP, non-synonymous | neutral variant [26] | damaging |
| rs28929472, Plowell | 9 | exon 3 | 93917039 | SNP, non-synonymous | deficiency variant [25] | damaging |
| rs1049800, Psaint albans | 1 | exon 3 | 93917038 | SNP, synonymous | neutral variant [25] | n/a |
| K259I | 6 | exon 3 | 93917030 | SNP, non-synonymous | deficiency variant [29] | damaging |
| T268I | 1 | exon 3 | 93917003 | SNP, non-synonymous | putative deficiency variant [21] | damaging |
| D270N^b^ | 1 | exon 3 | 93916998 | SNP, non-synonymous | putative deficiency variant [21] | damaging |
| rs200945035, Etokyo | 1 | exon 5 | 93914721 | SNP, non-synonymous | neutral variant [22] | damaging |
| rs61761869, Mwurzburg | 5 | exon 5 | 93914619 | SNP, non-synonymous | deficiency variant [28] | damaging |

Abbreviations: AAT, alpha1-antitrypsin; MAF, minor allele frequency; SNP, single nucleotide polymorphism; n/a, not applicable.

Chromosomal position is based on reference panel, NCBI build 36.3.

^a^ Number of participants carrying the variant heterozygously.

^b^ This individual also had the F-variant (rs28929470).
